# Interplay between Two-Component Regulatory Systems Is Involved in Control of Cupriavidus metallidurans Metal Resistance Genes

**DOI:** 10.1128/jb.00343-22

**Published:** 2023-03-09

**Authors:** Cornelia Große, Judith Scherer, Grit Schleuder, Dietrich H. Nies

**Affiliations:** a Martin-Luther-University Halle-Wittenberg, Institute for Biology/Microbiology, Molecular Microbiology, Halle, Germany; NCBI, NLM, National Institutes of Health

**Keywords:** *Cupriavidus metallidurans*, *Escherichia coli*, copper, two-component regulatory systems, zinc

## Abstract

Metal resistance of Cupriavidus metallidurans is based on determinants that were acquired in the past by horizontal gene transfer during evolution. Some of these determinants encode transmembrane metal efflux systems. Expression of most of the respective genes is controlled by two-component regulatory systems composed of a membrane-bound sensor/sensory histidine kinase (HK) and a cytoplasmic, DNA-binding response regulator (RR). Here, we investigated the interplay between the three closely related two-component regulatory systems CzcRS, CzcR_2_S_2_, and AgrRS. All three systems regulate the response regulator CzcR, while the RRs AgrR and CzcR_2_ were not involved in *czc* regulation. Target promoters were *czcNp* and *czcPp* for genes upstream and downstream of the central *czc* gene region. The two systems together repressed CzcRS-dependent upregulation of *czcP-lacZ* at low zinc concentrations in the presence of CzcS but activated this signal transmission at higher zinc concentrations. AgrRS and CzcR_2_S_2_ interacted to quench CzcRS-mediated expression of *czcNp-lacZ* and *czcPp-lacZ*. Together, cross talk between the three two-component regulatory systems enhanced the capabilities of the Czc systems by controlling expression of the additional genes *czcN* and *czcP*.

**IMPORTANCE** Bacteria are able to acquire genes encoding resistance to metals and antibiotics by horizontal gene transfer. To bestow an evolutionary advantage on their host cell, new genes must be expressed, and their expression should be regulated so that resistance-mediating proteins are produced only when needed. Newly acquired regulators may interfere with those already present in a host cell. Such an event was studied here in the metal-resistant bacterium Cupriavidus metallidurans. The results demonstrate how regulation by the acquired genes interacts with the host’s extant regulatory network. This leads to emergence of a new system level of complexity that optimizes the response of the cell to periplasmic signals.

## INTRODUCTION

Cupriavidus metallidurans strain CH34 is a metal-resistant bacterium that is also able to grow facultatively as a chemolithoautotroph or mixotroph with molecular hydrogen as the source for energy and redox equivalents (1, 2). Initially isolated in Mol, Belgium, at the end of the 1970s from a zinc decantation tank (3, 4), it has been kept since then in pure culture in several laboratories around the world. Natural environments of *C. metallidurans* are metal-rich soils with a possible geochemical source of molecular hydrogen, such as auriferous soils in Australia, serpentine soils in New Caledonia, or deserts with ample amounts of zinc (5–11). The genome of *C. metallidurans* comprises a chromosome, a chromid, and two large plasmids. All contain numerous genomic islands (1, 12, 13), which indicates that plasmid- and island-carrying genes were obtained by this bacterium during its evolution.

Metal resistance is based on the presence of inner membrane and transenvelope efflux pumps for transition metal cations and other metal resistance determinants (14). These determinants are mainly located on horizontally transmittable elements (13, 15). The set of genes allowing chemolithoautotrophic growth, including those encoding a membrane-bound and a soluble hydrogenase, as well as the enzymes of the Calvin cycle, is located on two genomic islands on the chromosome (15). The central features of this bacterium, essential to thrive in its ecological niche, were all obtained by horizontal gene transfer.

**FIG 1:**
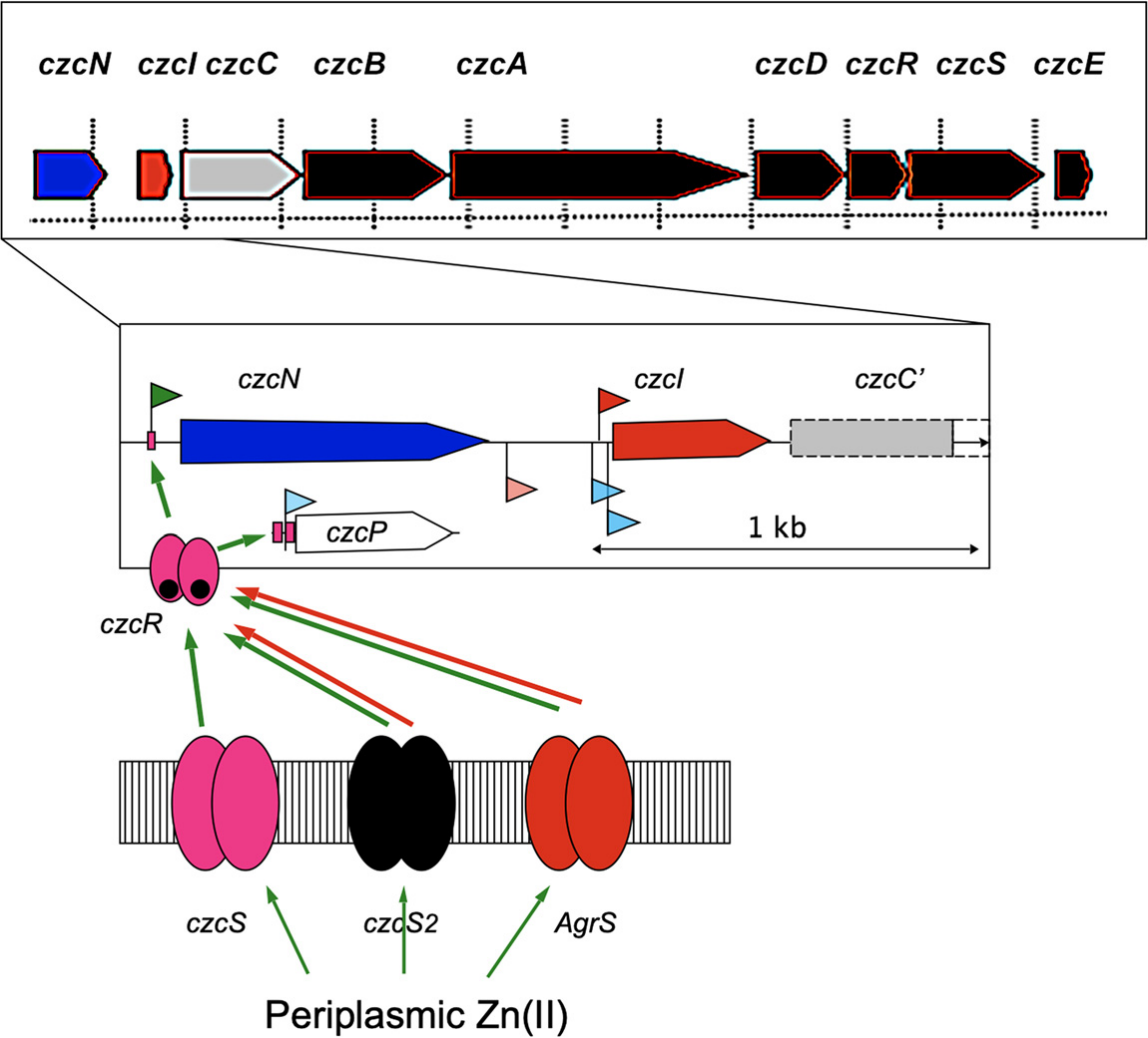
Schematic of the operon being studied. On top, the *czcNICBADRSE* region is shown with vertical size markers of 1 kb. Below, a magnification of the *czcNIC* region up to the middle of *czcC* is given. The open reading frame Rmet_6485 and *czcM* are omitted, but *czcP*, which is located further downstream of *czcE*, is shown (see Fig. S1 in the supplemental material). Flags indicate transcriptional start sites (29), RpoD-dependent promoters are in red, non-RpoD-dependent promoters are in blue, and a newly annotated non-RpoD promoter upstream of *czcN* is in green. Magenta bars upstream of *czcN* and *czcP* indicate one or two binding sites of CzcR, respectively. This response regulator essential for transcription initiation is shown as a phosphorylated (black dots) dimer (magenta) below *czcN* and *czcP*. It interacts with the membrane-bound histidine kinases CzcS (magenta), CzcS_2_ (black), and AgrS (red), which sense periplasmic zinc ions. Green arrows indicate activation; red arrows indicate inhibition.

The horizontally acquired metal resistance determinants contain paralogs for inner membrane and transenvelope efflux systems (16–18). Expression of the paralogous genes may be controlled by different regulatory circuits. For instance, the three genes *zntA*, *cadA*, and *pbrA*, encoding P_IB2_-type ATPases that export Zn(II), Cd(II), and Pb(II), respectively, are under the control of their own specific MerR-type regulators *zntR*, *cadR*, and *pbrR*, respectively (19–22). Of the 10 determinants encoding transenvelope efflux systems for divalent transition metal cations, which are composed of an inner membrane component of the RND (resistance nodulation protein family), an outer membrane factor, and a connecting membrane fusion (MFP) or adapter protein, many are inactivated by deleterious mutations of the central RND- or MFP-encoding genes, which identifies them as recessive determinants (15). The dominant determinants are the plasmid pMOL30-localized *czc* resistance determinant for cobalt, zinc, and cadmium resistance (Fig. 1) and *cnr* on pMOL28, which determines cobalt and nickel resistance.

Under laboratory conditions, the genome of *C. metallidurans* CH34 remains stable provided that the bacterium is kept under selection pressure alternating between high-Zn(II)-containing and high-Ni(II)-containing mineral salts medium. When the selection pressure is omitted, or the two plasmids are cured, the genome starts to deteriorate. Some genomic islands become deleted, for instance, those encoding the hydrogenases and the Calvin cycle enzymes. Surprisingly, most recessive metal resistance determinants are maintained, although their central components had been inactivated by mutation (15). This indicates that some of their genes may be required in the natural environment and under laboratory conditions. This appears to be the case for genes encoding membrane fusion proteins, outer membrane factors, and two-component regulatory systems on these recessive determinants, which were expressed under nonchallenging conditions or upregulated under challenging conditions. Could it be that metal resistance in *C. metallidurans* is a mosaic phenotype mediated by the dominant metal resistance determinants that interact with a few genes from the otherwise inactivated recessive determinants (15)? In this way, heterologous transenvelope efflux systems may exist or indeed cross talk between two-component regulatory systems could be involved, as has been described before in Escherichia coli (23). The aim of this study is to test this hypothesis.

Two-component regulatory systems are usually composed of a membrane-bound sensor(y) histidine kinase (HK) and cytoplasmic DNA-binding response regulator (RR), which is phosphorylated upon a signal being sensed by the HK (24). The RR dimerizes within seconds after the HK has received the stimulus and activates transcription (25). In *C. metallidurans*, the dominant *czc* resistance determinant (Fig. 1 and see Fig. S1 in the supplemental material) encodes the HK CzcS and the RR CzcR. The genes *czcRS* are part of the predicted operon Op1819f_1 *czcCBADRSE* on plasmid pMOL30, which is transcribed together with the *czcI* gene upstream of *czcC* from three transcriptional start sites (TSSs), one reliant on the housekeeping sigma factor RpoD, the other two not; 4 weak TSSs upstream of *czcI* and within the operon also exist. CzcD is a member of the CDF (cation diffusion facilitator) family of inner membrane secondary metal efflux systems and influences together with the periplasmic zinc- and copper-binding protein CzcE via CzcS *czc* expression (26–28). CzcI quenches the activity of the CzcCBA pump to prevent “overpumping” of essential metal cations such as Co(II) (22).

Many *czc* transcripts have been identified (Fig. S1), for instance, a *czcI* monocistronic message, *czcICBA*, and *czcDRS* (27, 29). The *czcCBADRSE* central *czc* region (Fig. 1; Fig. S1) is also expressed under nonchallenging conditions, and most genes are additionally upregulated under metal stress. The gene *czcN* upstream and *czcP*, coding for a zinc-exporting P_IB4_-type ATPase, downstream of the central *czc* determinant and separated from it by a transposon insertion were only weakly expressed under nonchallenging conditions (nucleotide activities per kilobase of exon model per million mapped reads [NPKM] value of 8 with 10 usually serving as a threshold; NPKM is a measure of the RNA abundance). Deletion of *czcP* decreases the zinc resistance of *C. metallidurans* in Tris-buffered mineral salts medium by one-third from an 50% inhibitory concentration (IC_50_) of 3.4 mM to 2.3 mM (30), but deletion of *czcS* or of *czcR* decreases resistance not at all (27). While *czcP* was upregulated under metal stress, *czcN* was not (15). Transcription of *czcN* and *czcP* depends on CzcR, and a binding site of CzcR had been identified at *czcNp* (27). Although the full picture of the regulation of *czc* by (i) RpoD, (ii) one or more non-RpoD-type sigma factors, and (iii) CzcRS is not known at this stage, expression of *czcN* and *czcP* cannot be upregulated without CzcR.

Intriguingly, genes of partially inactivated, recessive metal resistance determinants that were retained by *C. metallidurans* in the natural environment and laboratory are membrane fusion proteins, outer membrane factors, and two-component regulatory systems (15). This suggests fine-tuning of the activity of the dominant determinants by periplasmic signals or a function of proteins located in this compartment. We selected CzcR as a target to ask if CzcR activity as a regulator of two *czc* promoters might be under the control of cross-talking histidine kinases, CzcS and another HK encoded by recessive determinants. To that end, two-component regulatory systems from *C. metallidurans* were selected to investigate a possible cross talk with CzcRS. The results demonstrated that a cross talk indeed exists. It influences CzcR activity, especially at low zinc concentrations, to quench expression of *czcP* for an additional zinc-exporting P_IB4_-type ATPase and of *czcN* upstream of the main *czc* determinant. This might be useful to decrease loss of the essential trace element zinc at low external zinc concentrations and explains why the recessive determinants were retained under environmental and laboratory conditions.

## RESULTS

### Selection of possible cross-talking two-component regulatory systems in *C. metallidurans*.

The genome of *C. metallidurans* contains 160 genes for response regulators or sensor proteins, forming 42 gene regions with at least one gene for a response regulator and one for a sensory histidine kinase in close proximity. Among the predicted proteins for 29 histidine kinases, clustering of these proteins from *C. metallidurans* with CusS from E. coli (red) and an adjacent triple group containing CzcS (orange) (see Fig. S2 in the supplemental material) suggested an involvement in upregulation of copper or zinc resistance genes, respectively (31). The CusS and the CzcS clusters were related to YedV from E. coli and two more HKs from *C. metallidurans*. Another group of 10 HKs around QseC from E. coli (green) contained ZneS from *C. metallidurans*, which is encoded as part of the *zne-zni* metal resistance determinant (15, 29). While some of these HKs and RRs were associated with the genes for active (CzcRS, ZneRS, ZneR_2_S_2_, ZniRS, CopRS, and CopR_2_S_2_; Table S1, genes on a light green field) resistance determinants, other were affiliated with inactive (CzcR_2_S_2_, HmzRS; light red field) genes for transmembrane metal efflux systems (1, 18, 29). Except for ZneR_2_S_2_, the genes for these eight systems responded to metal stress (response of ≥5; Table S1). CzcRS, CopR_2_S_2_, HmzRS, Rmet_5797/98, and ZniRS were also expressed under nonchallenging conditions with an NPKM value of ≥10 for both genes (encoding the HK and RR), indicating a housekeeping function. ZneR_2_S_2_, not responding to metal stress, was also expressed in nonchallenged cells. Other HKs encoded in metal resistance determinants such as ZneS_2_ and ZniS were not closely related to CzcS from *C. metallidurans* (Fig. S2).

A multiple alignment of the RRs of metal resistance determinants in *C. metallidurans* displayed a related pair, CopR_1_ and CopR_2_ (red), while CzcR, CzcR_2_, and Rmet_1751 were less related to the CopR pair (Fig. S3, orange). Other RRs clustered around ZneR (green). The two-component regulatory systems in *C. metallidurans* with the highest probability for a metal-dependent cross talk were the systems CzcRS, CzcR_2_S_2_, and Rmet_1751/Rmet_1752. The respective three HKs form a cluster of 3 proteins (Fig. S2, orange) distant from HKs involved in copper-dependent regulation (Fig. S2, red). The two-component regulatory system Rmet_1751/Rmet_1752 is involved in a spontaneous development of silver resistance in *C. metallidurans* (32) and was renamed AgrRS. Consequently, possible cross-talking two-component regulatory systems in *C. metallidurans* involved in fine-tuning of zinc homeostasis could be CzcRS, CzcR_2_S_2_, and AgrRS. These three systems were selected for further investigation.

### Transcription of the *czc* region.

Transcriptional organization of the *czc* region on plasmid pMOL30 has been extensively studied (15, 26, 27, 29, 33, 34), and the data are summarized in Fig. 1 and Fig. S1. Several promoters upstream of *czcI* are responsible for transcription of *czcICABDRS*. The determinant is expressed even in nonchallenged cells and responds strongly to changes in metal availability.

Transcription of *czcN* in comparison to the other *czc* genes was analyzed in detail using reverse transcriptase quantitative PCR (qRT-PCR) in the parent strain AE128, a derivative of CH34 with only plasmid pMOL30 (2), and its isogenic Δ*czcR*, Δ*czcS*, and Δ*czcP* mutants (Table 1). The *czcN* gene did not respond to metal stress in gene array experiments (Fig. S1); however, in these experiments, *C. metallidurans* cells were challenged with a metal mixture containing 30 μM (each) Co(II), Ni(II), Cu(II), Zn(II), and Cd(II) (33). This may have been too low a zinc concentration to induce upregulation of *czcN*. Instead of the metal mix, the cells were confronted with 300 μM Zn(II), and a qRT-PCR experiment was performed. At this concentration, *czcN* and the other tested *czc* genes were clearly upregulated in the presence of Zn(II) in *C. metallidurans* AE128 (Table 1).

**TABLE 1:** Transcription of genes of the *czc* operon after induction with 300 μM Zn(II)^*a*^

Relevant genotype	Transcription of gene:						
	*czcN*	*czcI*	*czcA*	*czcD*	*czcR*	*czcE*	*rpoZ*
AE128	18.1 ± 4.5	9.3 ± 0.9	16.3 ± 5.6	17.0 ± 10.8	11.5 ± 3.1	7.8 ± 3.8	0.76 ± 0.12
Δ*czcR*	1.5 ± 0.2	34.1 ± 6.9	50.8 ± 13.6	37.1 ± 11.5	ND	11.0 ± 1.2	1.08 ± 0.23
Δ*czcS*	1.6 ± 0.4	23.1 ± 1.8	31.9 ± 9.0	23.6 ± 6.7	20.4 ± 3.9	21.3 ± 11.8	1.27 ± 0.27
Δ*czcP*	16.6 ± 2.1	14.8 ± 9.6	44.6 ± 25.5	29.3 ± 19.9	29.0 ± 9.0	9.7 ± 2.1	1.46 ± 0.93

a *C. metallidurans* strains AE128(pMOL30), DN178(pMOL30-10, Δ*czcR*), DN179(pMOL30-11, Δ*czcS*), and DN493(pMOL30, Δ*czcP*) were treated for 10 min with 300 μM Zn(II) or without added metal, and RNA was isolated. This zinc concentration yields the best transcript response for both regions, *czcCBA* and the genes upstream of *czcC* (26, 34). Four quantitative RT-PCR determinations and two independent cultivations were performed. The quotients induced/not induced were determined. Quotients of ≥2 are underlined. The *rpoZ* gene, coding for a subunit of the RNA polymerase, was used as an internal standard. ND, not done.

In contrast to the parental strain AE128, *czcN* was not upregulated by treatment for 10 min with 300 μM Zn(II) in the Δ*czcR* and the Δ*czcS* mutants (Table 1), while all other *czc* genes were still upregulated in both mutants. In the Δ*czcP* mutant, which does not produce the zinc-exporting P_IB4_-type ATPase CzcP (30), *czcN* was again upregulated. Expression of *czcN* was clearly CzcRS dependent, but the expression of other *czc* genes in operon Op1819f_1 from *czcI* to *czcE* was not, because these genes rely on the *czcI* promoters (Fig. 1). Expression of *czcN* occurred only at high zinc concentrations.

### Control of expression of *czcPp-lacZ* on plasmid pMOL30.

**FIG 2:**
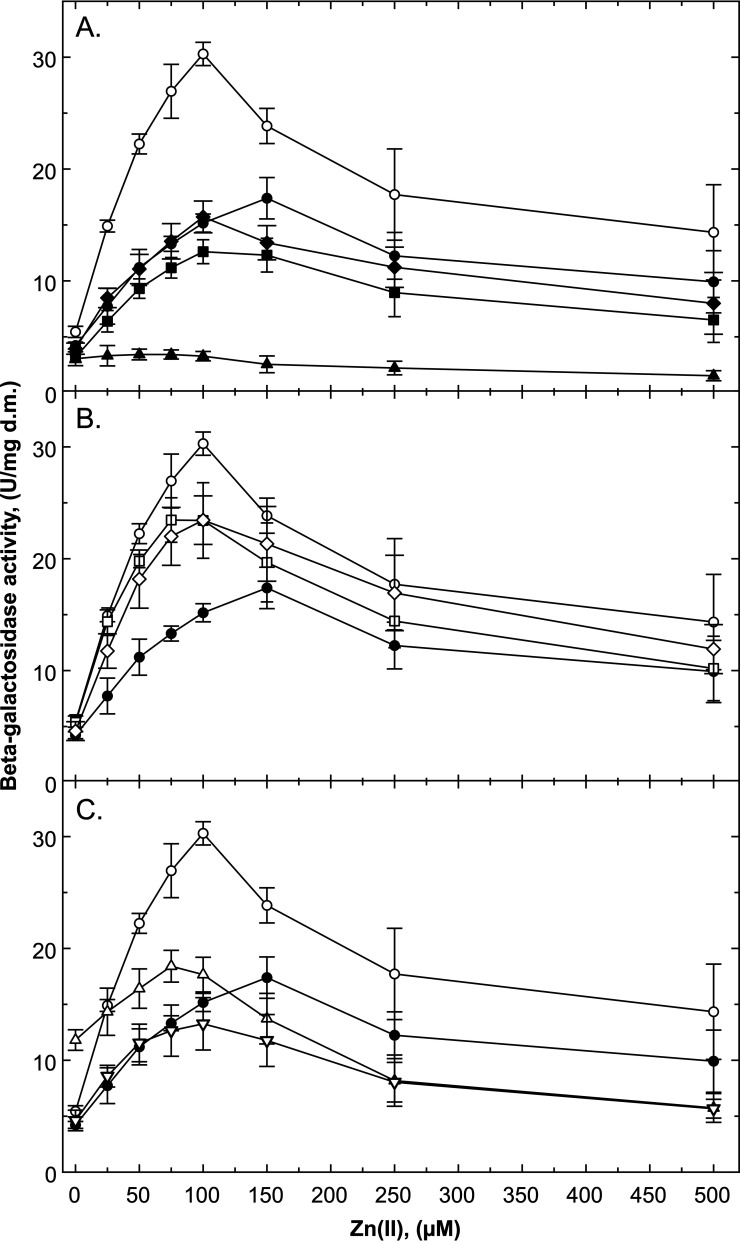
Concentration-dependent increase in expression of *czcP-lacZ* on plasmid pMOL30. The cells were incubated with various zinc concentrations for 3 h, and the specific beta-galactosidase activity was determined. The strains carrying the *czcP-lacZ* fusion derived from AE128 parent (closed circles) and DN179 (Δ*czcS*, open circles) are shown in all panels. (A) Single mutants DN178 (Δ*czcR*, closed triangles), DN572 (Δ*agrRS*, closed squares), and DN573 (ΔczcR_2_S_2_, closed diamonds). (B) Δ*czcS* double mutants DN575 (Δ*czcS* Δ*agrRS*, open squares) and DN576 (Δ*czcS*, Δ*czcR_2_S_2_*, open diamonds). (C) Double mutant DN574 (Δ*agrRS* Δ*czcR2S2*, open triangles) and triple mutant DN577 (Δ*czcS* Δ*czcR_2_S_2_* Δ*agrRS*, open inverted triangles). *n* = 5 for Δ*czcR*; *n* > 6 for all others; deviations shown. d.m., dry mass.

The *lacZ* gene was inserted downstream of the *czcP* gene on plasmid pMOL30, which has a copy number of 1 compared to the chromosome (35). Time- and concentration-dependent upregulation of *czcP-lacZ* expression was determined (Table 2, Fig. 2, and Fig. S4). As previously observed (30), upregulation of *czcP* was strictly zinc and *czcR* dependent (Fig. 2). First, expression of *czcP* essentially required the zinc-dependent activation of CzcR, probably by phosphorylation (24). Second, none of the other RRs in *C. metallidurans* related to CzcR (Fig. S3) were able to substitute for CzcR. These RRs were not relevant for further discussion.

In the Δ*czcS* mutant, expression of *czcP-lacZ* was twice as strong as that in the parent. In the Δ*czcS*, Δ*agrRS*, and Δ*czcR_2_S_2_* strains, the maximum reporter activity was reached at 100 μM Zn(II), and in the parent, it was reached at 150 μM (Fig. 2A). The time-dependent increase in reporter activity in the Δ*czcS* mutant was 5.2 times higher than in the parent, but the regulatory response displayed a lag of 34 min (Table 2). Other HKs were able to phosphorylate CzcR, which took about 30 min before activation of transcription began. This lag also explains why for the other CzcR-regulated gene, *czcN*, no upregulation was observed in the Δ*czcS* mutant when analyzed by qRT-PCR after only 10 min (Table 1).

**TABLE 2:** Time-dependent activation of *czcPp-lacZ* expression on plasmid pMOL30^*a*^

Strain	Genotype	Without Zn(II)				200 μM Zn(II)				*Q*(*b*)
		RegCoff. (%)	*a* (U/mg)	*b* (U/mg/min)	*t*(lag) (min)	RegCoff. (%)	*a* (U/mg)	*b* (U/mg/min)	*t*(lag) (min)	
AE128	Parent	96.9	20.0 ± 1.5	−0.025 ± 0.003	47	99.8	20.0 ± 1.5	0.078 ± 0.002	<0	1.00
DN178	Δ*czcrR*	97.6	18.7 ± 0.9	−0.020 ± 0.002	<0	99.0	18.7 ± 0.9	−0.022 ± 0.002	6	**−0.28**
DN179	Δ*czcS*	30.2	22.4 ± 1.1	−0.002 ± 0.002	<0	99.9	22.4 ± 1.1	0.405 ± 0.010	34	**5.20**
DN572	Δ*agrRS*	97.7	23.0 ± 1.6	−0.031 ± 0.003	6	97.1	23.0 ± 1.6	0.058 ± 0.007	<0	**0.75**
DN573	Δ*czcR_2_S_2_*	88.6	23.0 ± 1.5	−0.014 ± 0.003	69	90.9	23.0 ± 1.5	0.047 ± 0.011	<0	**0.61**
DN574	Δ*agrRS ΔczcR_2_S_2_*	95.9	42.5 ± 2.1	−0.023 ± 0.003	<0	92.6	42.5 ± 2.1	0.094 ± 0.019	3	1.21
DN575	Δ*czcS* Δ*agrRS*	6.7	24.2 ± 0.8	0.002 ± 0.006	596	99.2	24.2 ± 0.8	0.305 ± 0.020	60	**3.92**
DN576	Δ*czcS* Δ*czcR_2_S_2_*	98.6	25.4 ± 1.4	−0.021 ± 0.002	<0	99.7	25.4 ± 1.4	0.363 ± 0.013	54	**4.66**
DN577	Δ*czcS* Δ*agrRS* Δ*czcR_2_S_2_*	91.2	27.0 ± 1.3	−0.025 ± 0.006	51	97.6	27.0 ± 1.3	0.261 ± 0.029	64	**3.35**

a The cells were incubated with or without 200 μM Zn(II), and the specific beta-galactosidase activity was measured after the indicated time (see Fig. S4 in the supplemental material). Compared to Table 1, the inducing zinc concentration was lowered from 300 μM to 200 μM because the response of the *czcPp-lacZ* fusion was already past the maximum at 300 μM zinc (Fig. 2). For the mean values from 6 experiments, a linear curve fitting was performed for all values at *t* ≥ 60 min to the function *y* = *a* + *b* × *t*. The regression coefficient, *a* values, and values for the slope *b* are indicated with the deviation values stemming from the curve fitting algorithm (pro Fit 7.0.19, www.quansoft.com). Moreover, the lag or liftoff time was calculated according to *t*(lag) = [*y*_(_*_t_*_= 0)_ − *a* + 60 × *b*]/*b*. The *Q*(*b*) value is the ratio of the *b* values of zinc-induced mutants divided by the *b* value of zinc-induced parent. It is shown in bold letters for *D* = (absolute difference in mean values divided by the sum of the deviations) > 1.

In the two double mutant Δ*czcS ΔagrRS* and Δ*czcS ΔczcR_2_S_2_* strains, the zinc-dependent expression level of *czcP-lacZ* was between those of the Δ*czcS* mutant and the parental strain AE128 (Fig. 2B). The time-dependent increase in reporter activity was 3.9- and 4.7-fold higher in these double mutants than in the parent (Table 2) but with a lag of 1 h. Expression of *czcP-lacZ* in the Δ*czcS* Δ*agrRS* Δ*czcR_2_S_2_* triple mutant was not higher but was on a similar level as the parent at zinc concentrations up to 75 μM and on a lower level at higher zinc concentrations (Fig. 2C). The increase in reporter activity in the triple mutant was 3.3-fold higher than in the parental strain, and the lag phase was again about 1 h (Table 2). This means that at least one other two-component system was able to activate CzcR. Second, AgrRS and CzcR_2_S_2_ were responsible for the high activation level of *czcP-lacZ* in the absence of CzcS. Third, activation of CzcR by the other systems was delayed while activation in the presence of CzcS was a rapid process (Table 2). Finally, CzcS_2_ and AgrS cooperated to reduce the lag time of activation by approximately 50%.

In the presence of CzcS, expression of *czcP-lacZ* in the Δ*czcR_2_S_2_* mutant was similar to that in the parental strain up to concentrations of 75 μM Zn(II) and on a slightly lower level at higher zinc concentrations, while that of the Δ*agrRS* mutant was lower at all concentrations than the parental strain (Fig. 2A). The increase in reporter activity was lower than in the parent, and no lag could be observed (Table 2). The increased expression observed in the Δ*agrRS* Δ*czcR_2_S_2_* double mutant was similar to that in the parental strain (Table 2) but started at a higher level in the zinc-dependent measurement when no zinc was added. This paralleled the expression profile measured for the parental strain up to 75 μM Zn(II) but reached a lower overall expression level at higher zinc concentrations (Fig. 2C). AgrRS and CzcR_2_S_2_ together repressed CzcRS-dependent upregulation of *czcP-lacZ* at low zinc concentrations in the presence of CzcS but activated this signal transmission at higher zinc concentrations. Both needed to interact for this purpose. In the absence of the one of the systems, and at low zinc concentrations up to 75 μM, CzcR_2_S_2_ alone had a small activating effect, which was observed only in the time-dependent experiments, while this effect was evident for AgrRS in the time- and the concentration-dependent experiments. AgrRS and CzcR_2_S_2_ thus interacted with CzcRS to control expression of *czcP* via CzcR.

### Expression of *czcNp-lacZ* cloned on plasmid pVDZ′2.

The DNA region between the 3′ end of *czcN* and the beginning of *czcI* includes several transcriptional start sites and a possible open reading frame, Rmet_6485, encoding a protein with unknown function (Fig. S1). Insertion of *lacZ* downstream of *czcN* might disturb expression of *czc* by causing deleterious effects. Consequently, *czcNp* was cloned upstream of a *lacZ* gene on plasmid pVDZ′2, a derivative of plasmid RP4/RK2, which should have a 3- to 5-fold-higher copy number than plasmid pMOL30 (36). Due to this copy number, titration of the regulators can occur, as has been shown to occur in the Fur titration assay (FURTA) (37). Thus, the beta-galactosidase activity should be higher than the fusion on plasmid pMOL30. The negative controls indeed showed a high expression level, even in the promoterless control. Again, no zinc-dependent upregulation occurred in the absence of CzcR (Fig. S5), as previously reported (26).

**FIG 3:**
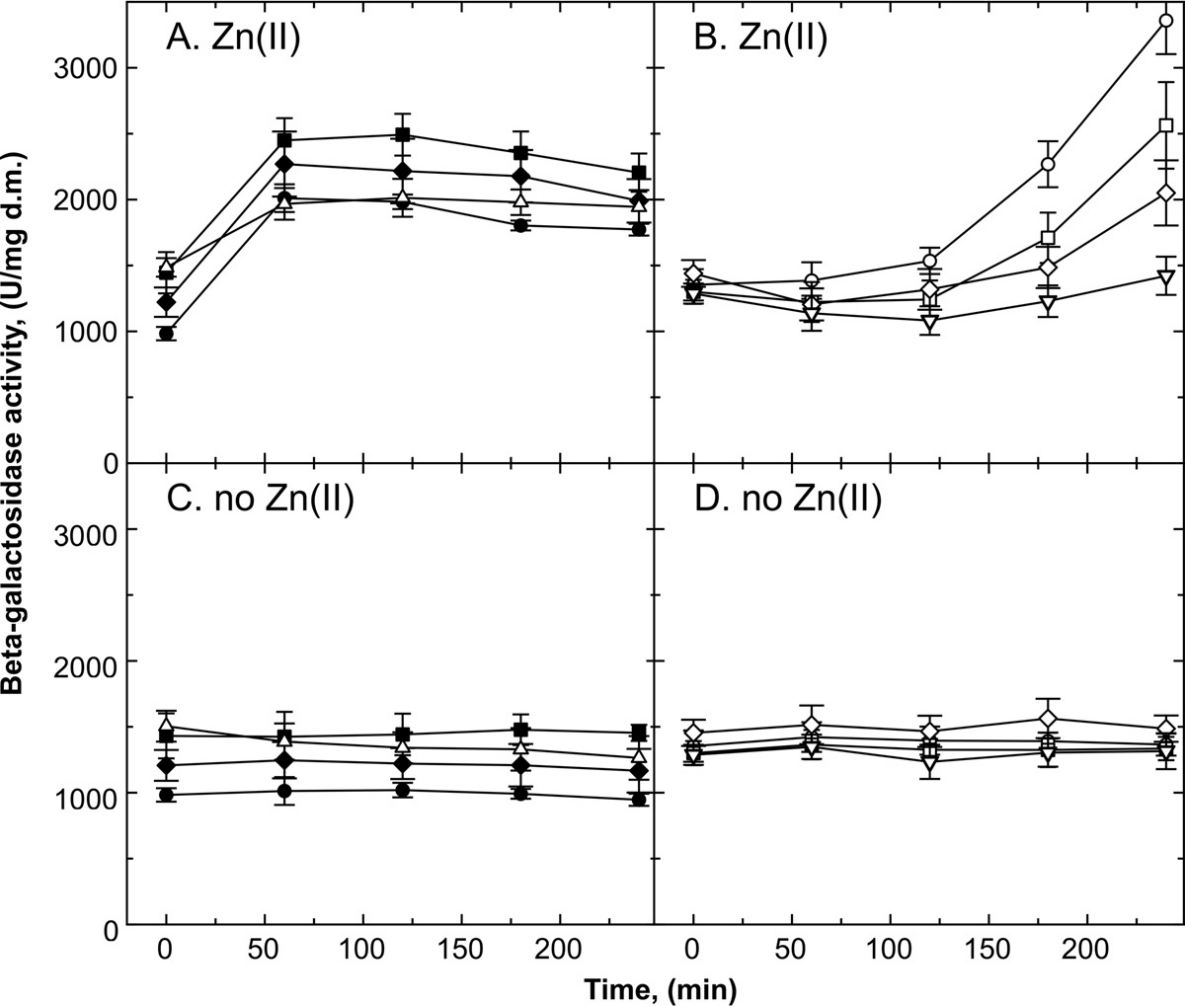
Time-dependent increase in expression of *czcNp-lacZ* on vector plasmid pVDZ′2. The cells were incubated with 750 μM Zn(II) (A and B) or without Zn(II) (C and D), and the specific beta-galactosidase activity was measured after the indicated time. An inducer concentration of 750 μM Zn(II) was used for *czcNp-lacZ* compared to *czcCp-lacZ* because *czcNp-lacZ* needed a higher zinc concentration than *czcCp-lacZ* for an optimal response. Shown are the strains carrying the *czcNp-lacZ* fusion on vector plasmid pVDZ′2 in AE128 parent (closed circles), DN179 (Δ*czcS*, open circles), DN572 (Δ*agrRS*, closed squares), DN573 (ΔczcR_2_S_2_, closed diamonds), DN574 (Δ*agrRS* Δ*czcR_2_S_2_*, open triangles), DN575 (Δ*czcS* Δ*agrRS*, open squares), DN576 (Δ*czcS*, Δ*czcR_2_S_2_*, open diamonds), or DN577 (Δ*czcS* Δ*czcR_2_S_2_* Δ*agrRS*, open inverted triangles). Strain DN178 (Δ*czcR*) is shown with other controls in Fig. S5 in the supplemental material. For the results shown in panel B, a polynomial curve fit to the function “*y* = *a* + *bt* + *ct*^2^” was performed (fitting coefficients 98.2% to 99.5%; see Table S2 in the supplemental material). The *a* values (1,354 ± 67 U/mg) and the *b* values (−4.82 ± 0.79 U/mg/min) were not different. The *c* value of strain DN179 (Δ*czcS*) was 3-fold (*D* = 1.82), that of strain DN575 (Δ*czcS* Δ*agrRS*) was 2.4-fold (*D* = 1.13), and that of strain DN576 (Δ*czcS*, Δ*czcR_2_S_2_*) was 1.8-fold (*D* = 0.73, not significant) higher than that of the triple mutant DN577. *n* > 6; deviations shown. d.m., dry mass.

The time-dependent expression of *czcNp-lacZ* in plasmid pVDZ′2 was strictly zinc dependent (Fig. 3). In the presence of CzcS (Fig. 3A), zinc-mediated upregulation of *czcNp-lacZ* expression in the Δ*agrRS* and the Δ*czcR_2_S_2_* single mutants paralleled the profile of the parental strain on a slightly higher expression level so that these systems alone activated CzcRS-mediated expression of *czcNp-lacZ* to a small degree. The Δ*agrRS* Δ*czcR_2_S_2_* double mutant started from a higher basic expression level but reached the expression level of the parental strain after 1 h. In the absence of zinc, AgrRS and CzcR_2_S_2_ interacted to quench CzcRS-mediated expression of *czcNp-lacZ*, similarly to *czcPp-lacZ*.

While in all single, double, and triple mutants bearing a Δ*czcS* mutation expression of *czcP-lacZ* on plasmid pMOL30 started after a lag and increased subsequently following a linear function (Table 2; Fig. S4), the zinc-dependent expression profiles of *czcNp-lacZ* on plasmid pVDZ′2 followed a polynomial function “*y* = *a* + *bt* + *ct*^2^” (Fig. 3B). The *a* and *b* coefficients of the four mutants were not different (Table S2) whereas the *c* values increased, compared to the triple mutant, 1.8-fold in the Δ*czcS ΔczcR_2_S_2_* mutant, 2.4-fold in the Δ*czcS ΔagrRS* mutant, and 3-fold in the Δ*czcS* single mutant. With this result in mind, the data from the *czcP-lacZ* fusion in the Δ*czcS* mutants were also fitted to a polynomial function (Table S2). Again, the *a* values of the four mutants were similar. The *b* values had large deviations, indicating that a linear function with a lag (Table 2) delivered a better modeling of the data than a polynomial function. The *b* value decreased from the Δ*czcS* mutant to the triple mutant in both models. The *c* values of the double and triple mutants were similar, the *c* value for the Δ*czcS* mutant being half of this value, reminiscent of the doubling of the lag from the single to the double and triple mutants. Since the first derivative of a second-grade polynomial function at time still depends on the time, both a polynomial fitting and a linear fitting with a lag described the data by different modeling approaches. These two sides of the same coin demonstrated that activation of CzcR by the other histidine kinases was a delayed process. Possible explanations could be a lower phosphorylation rate of CzcR by CzcS_2_ and AgrS than by CzcS or an upregulation of *czcR_2_S_2_* and *agrRS* expression that had to occur before CzcR could be efficiently activated.

**FIG 4:**
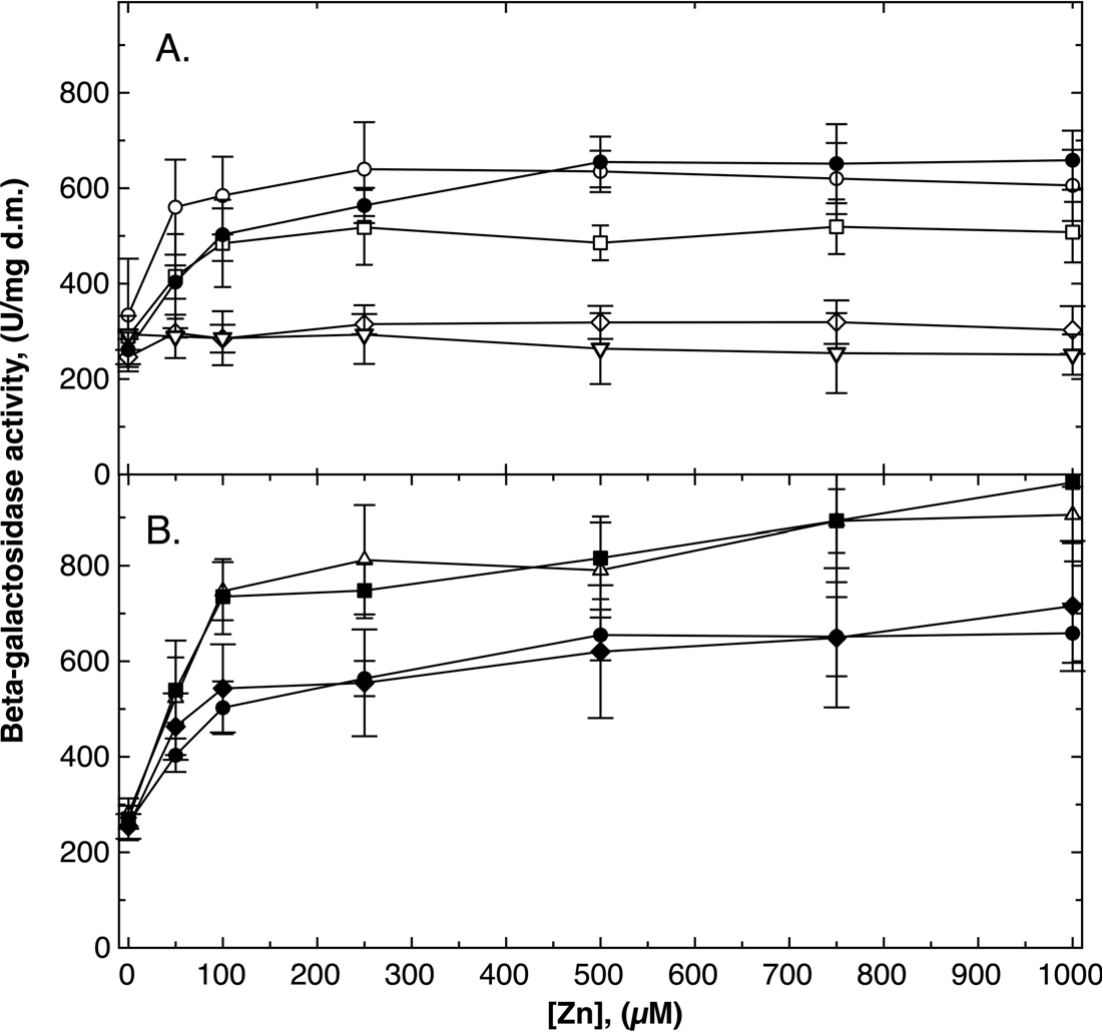
Influence of Δ*czcR_2_S_2_* and Δ*agrRS* deletions on control of the zinc-induced expression of a *czcNp-lacZ* reporter gene fusion on a medium-copy-number plasmid. A copy of plasmid pVDZ′2 containing a *czcNp-lacZ* promoter fusion to the *lacZ* reporter gene was conjugated into mutant strains of *C. metallidurans* AE128(pMOL30), and induction of the reporter by increasing zinc concentrations was measured. (A) Wild-type control AE128(pMOL30) (closed circles) and the mutant strains DN179 (Δ*czcS*, open circles), DN575 (Δ*czcS* Δ*agrRS*, open squares), DN576 (Δ*czcS*, Δ*czcR_2_S_2_*, open diamonds), and DN577 (Δ*czcS* Δ*czcR_2_S_2_* Δ*agrRS*, open inverted triangles). (B) Wild-type control AE128(pMOL30) (closed circles), DN572 (Δ*agrRS*, closed squares), DN573 (ΔczcR_2_S_2_, closed diamonds), and DN574 (Δ*agrRS* Δ*czcR_2_S_2_*, open triangles). Three biological repeats; deviation bars shown. d.m., dry mass.

The time-dependent *czcNp-lacZ* experiments indicated also a higher contribution of CzcR_2_S_2_ to expression in the absence of CzcS than of AgrRS, while that of the third, unknown system was minor (Fig. 3B). This was also demonstrated by the concentration-dependent expression profiles of *czcNp-lacZ* on plasmid pVDZ′2 (Fig. 4). In the Δ*czcS* single mutant (Fig. 4A), the expression level was higher than that of the parental strain at zinc concentrations up to 200 μM. No upregulation occurred in the Δ*czcS ΔagrRS ΔczcR_2_S_2_* triple mutant. The level of the Δ*czcS ΔczcR_2_S_2_* mutant was barely above the level of the triple mutant while that of the Δ*czcS agrRS* mutant was between those of the triple mutant and the parental strain. AgrRS and CzcR_2_S_2_, together, were able to activate *czcNp-lacZ* expression, and the contribution of CzcR_2_S_2_ was higher than that of AgrRS (Fig. 4A). In the presence of CzcS, the expression profile of the Δ*czcR_2_S_2_* mutant was similar to that of the parent and that of the Δ*agrRS* mutant was similar to that of the Δ*agrRS ΔczcR_2_S_2_* double mutant. In both Δ*agrRS*-containing strains, the *czcNp-lacZ* expression level was higher than that of the parent (Fig. 4B). In the presence of CzcS, AgrRS quenched zinc-dependent *czcNp-lacZ* expression while CzcR_2_S_2_ did not interfere. In the absence of CzcS, AgrRS and CzcR_2_S_2_ interacted to mediate expression of *czcNp-lacZ* after a lag period with a stronger contribution coming from CzcR_2_S_2_ than from AgrRS.

Expression of *czcIp-lacZ* on plasmid pVDZ′2 was on an even higher level of reporter activity than that of *czcNp-lacZ* (Fig. S6). The differences between the mutants and their parent were small with a slightly higher expression level of the Δ*czcS* mutant at low zinc concentration than of the parent and a lower expression level of the two double mutants and the triple mutant carrying a Δ*czcS* mutation. This finding was in agreement with an observed upregulation of the genes from *czcI* to *czcE* (Table 1) and all *czc* structural genes (27) in the Δ*czcS* mutant. It also indicated that AgrRS and CzcR_2_S_2_ interacted to influence regulation of expression of the *czcI* promoter region with its minimum of 4 transcriptional start sites (Fig. 1; Fig. S1).

### Identification of the *czcN* promoter.

Previously identified possible transcriptional start sites for *czcN* are located 643 bp, 834 bp, 844 bp, and 1,395 bp upstream of *czcN* (29). This is further upstream of *czcN* than the beginning of the 246-bp fragment that was cloned in plasmid pVDZ′2, which displayed a clear metal-dependent upregulation of the *lacZ* reporter (Fig. 3 and 4) (26). A transcriptional start site 70 bp upstream of *czcN* (open reading frame 74211.74861, NC_007971.2), TSS_74141+4 (Table S1, rank 3714) (29) was annotated to the incorrect open reading frame due to repeated changes in the annotation of the *C. metallidurans* genome. This TSS was not assigned to the RpoD sigma factor. Its activity score under nonchallenging conditions is 37.3 ± 9.3 (29) and represents a weak activity. In a new RNA sequencing and TSS determination experiment, *C. metallidurans* CH34 was treated with a modified metal mixture (33) and either with 50 μM EDTA or with no addition. RNA was isolated and used to determine the transcriptional start sites. In this experiment, the score of TSS_74141+4 in RNA from metal-challenged cells was 60,069 ± 10,166, in nonchallenged cells it was 232 ± 42, and in EDTA-treated cells it was 236 ± 43. There was no upregulation of *czcNp* in the presence of the metal chelator EDTA but a 259-fold upregulation under conditions of metal stress. The 246-bp fragment thus carries the *czcNp* promoter, which had previously not been identified.

## DISCUSSION

### Accumulation of genes by the *czc* metal resistance determinant on plasmid pMOL30 mediated an increase in complexity.

Plasmid pMOL30 of *C. metallidurans* strain CH34 is a horizontally acquired replicon that provides sophisticated copper *cop_1_* and cobalt-zinc-cadmium resistance *czc* determinants to its host, in addition to another mercury (*mer*), a lead (*pbr*), and an inactivated nickel-cobalt-cadmium (*ncc*) resistance determinant (1, 38–40). The *cop_1_* and *czc* determinants contain more genes than paralogous determinants on the chromosome and chromid. Plasmid pMOL30 will be maintained by *C. metallidurans* only if the plasmid-carried determinants provide a better function than the chromosomal or chromid paralogs, so that the plasmid-carried determinants become dominant over the others.

The paralogous chromid-carried *czc_2_* determinant of *C. metallidurans* displayed clear signals of a recessive determinant (15). Originally, a *zntA*<>*czcI_2_C_2_B_2_A_2_*, *czcR_2_S_2_* determinant as found in related bacterial strains (11, 16) was interrupted within the *czcB_2_* genes. Subsequently, the two parts of the determinant were separated from each other on the chromid by a rearrangement of this replicon. ZntA is a P_IB2_-type ATPase and the major zinc-exporting inner membrane efflux system of *C. metallidurans* (30, 41). The *zntA* gene is under the control of an RpoD-dependent promoter and the MerR-type regulator ZntR (22). This fate of *czc_2_* as a result of acquisition of plasmid pMOL30 with its *czc* determinant by *C. metallidurans* indicates that *czc* provides a function to *C. metallidurans* that was superior to that mediated by *czc_2_*. The additional benefit should be encoded by those genes of *czc* that *czc_2_* does not contain, namely, *czcD*, *czcE*, *czcJ*, *czcP*, and *czcN*. Among these beneficial genes, *czcP* and *czcN* are expressed under the control of CzcRS and its cross-talking partners, while *czcD* and *czcE* flank *czcRS* and influence the activity of CzcRS (26).

The central *czcICBADRSE* region of *czc* (Fig. 1) is upregulated when the cells are challenged by high metal concentrations, with zinc being the best inducer, followed by cobalt and cadmium (26, 34). Depending on the method used, *czc* allows an IC_50_ of zinc of about 3.4 mM in liquid culture (30) or a MIC of 12 mM on solid medium (2). On the other hand, *czc* is expressed even under nonchallenging conditions in Tris-buffered mineral salts medium (see Fig. S1 in the supplemental material). The zinc content of this medium is 200 nM (42). Despite this low zinc concentration and expression of *czc* in these cells, they are able to obtain sufficient zinc to grow. This changes when the central zinc importer ZupT is deleted. In this case, pMOL30 is rapidly cured from the cells (42). A forced expression of *czcCBA* from a plasmid in *trans* results in a disappearance of the central CzcA protein from the cells despite the presence of its mRNA. This suggests either that translation of the mRNA is impeded or that degradation of CzcA occurs. This indicates that the pMOL30-carried *czc* determinant has the ability to interact indirectly with ZupT and the other components of the Zur regulon (43–45) so that zinc homeostasis is maintained from 200 nM to the lower-millimolar range of zinc concentrations, covering a 10,000-fold difference when considering only the zinc concentration. Additionally, *czc* mediates homeostasis of the minor bioelements cobalt and the toxic-only cadmium. All the additional genes of *czc* could be involved in this process, bringing a greater advantage to the cells than *czc_2_*. Since *czc* mediates resistance to three metal cations, one toxic only and two essential but toxic, expression of *czc* also needs a high level in sophistication of its regulation.

Under nonchallenging conditions, four transcriptional start sites were identified 284 bp, 97 bp, 53 bp, and 31 bp upstream of *czcI* (29). The highest-abundance mRNAs initiated transcription at the 53-bp position, and this start site was also identified previously by primer extension (27). The associated promoter displayed a medium-strong consensus sequence for the housekeeping sigma factor RpoD. The abundance of mRNAs with 5′ ends at the 97-bp and 31-bp upstream positions was about 10% of that of the 53-bp 5′ mRNA, and both appear not to be RpoD promoters. Other sigma factors contribute to *czcI* expression. The remaining start site most distal to *czcI* had a 2% abundance but displayed a strong consensus motif for RpoD-dependent promoters.

Three additional start sites within the *czcICBADRSE* region contributed to a low abundance (2% to 3% of that from the 53-bp position) of mRNAs with 5′ ends at these sites, which were located 32 bp upstream of *czcA*, 1,003 bp upstream of *czcD*, and 37 bp upstream of *czcE*, respectively. None was an obvious RpoD-dependent promoter (29), so that other sigma factors might initiate transcription of parts of *czc* from these promoters (Fig. S1, white arrowheads). A 5′ mRNA end 223 bp upstream of *czcC* was identified by primer extension but not by transcriptome sequencing (RNA-Seq) under nonchallenging conditions (27, 29). Provided such a start site cannot be found under challenging conditions, the appearance of *czcI* and *czcICBA* mRNAs in Northern blots (27) and the decrease in mRNA abundance from *czcI* to *czcC* (Fig. S1) would indicate an mRNA cleavage site 223 bp upstream of *czcC*, which is within the *czcI* gene. No transcript continued between *czcA* and *czcD*, or between *czcS* and *czcE* in zinc-treated cells, in agreement with stem-loop structures downstream of *czcA* and *czcS* that may act as transcriptional terminators (27). The abundance of mRNAs starting 1,003 bp upstream of *czcD* and 37 bp upstream of *czcE* was 36 ± 4 and 26 ± 5 in nonchallenged cells, respectively, which fits the NPKM values for *czcD* of 26.3 ± 0.6 and for *czcE* of 26.3 ± 3.1. The promoter sequences associated with both start sites were clearly not RpoD-dependent promoters (29), so that *czcDRS* and *czcE* were expressed as a tricistronic and monocistronic message, respectively, under the control of one or two non-RpoD-dependent RNA polymerase holoenzymes. This indicates that the predicted operon Op1819f_1 (Fig. S1) contains in fact three transcriptional units, *czcCBA*, *czcDRS*, and *czcE*.

Upstream of the *lacZ* reporter gene on plasmid pVDZ′2, the *czcI* promoter region mediated a high beta-galactosidase activity, which is upregulated about 30% by increasing zinc concentrations. CzcS and CzcR are not needed for upregulation (26), and the influence of CzcS and the other two histidine kinases was small (Fig. S6). Consequently, CzcR and CzcS do not control expression of the central *czcICBA* gene region. Transcription of this core part of *czc* is guaranteed by the housekeeping sigma factor RpoD and additionally by at least one non-RpoD sigma factor. Instead, CzcR and CzcS influence expression of flanking genes *czcN* and *czcP*. On the next level, expression of *czcR* and *czcS*, as part of the tricistronic *czcDRS* mRNA, is under the control of a non-RpoD sigma factor (Fig. S7, fields with a red or blue surrounding for exclusively non-RpoD control or control by both, non-RpoD and RpoD, respectively), for instance, the RpoH heat shock factor, the RpoS stationary-phase factor, or one of the 11 sigma factors of the extracytoplasmic function (ECF) family (33, 46, 47). Since the consensus sequences for all these sigma factors have not yet been identified, anything between one non-RpoD sigma factor controlling both non-RpoD-dependent *czcI* promoters, *czcDp* and *czcEp*, and four sigma factors controlling each one of these promoters can be assumed. This third pillar of metal homeostasis (33) regulates expression of *czcICBA*, of *czcDRS*, and of *czcE*, and subsequently, CzcD, CzcR, CzcS, and CzcE control transcription initiation of *czcN* and *czcP*. Finally, the cross talk between the two-component regulatory systems was now added to the model (Fig. S7).

### Function of the products of the *czcICBA* gene region.

The large transmembrane-spanning CzcCBA protein complex is at the core of the Czc system. There is clear evidence that the RND protein CzcA and the related copper transporter CusA transport their substrates *in vitro* across a membrane that would correspond to the inner or cytoplasmic membrane (48–50). But there is also accumulating evidence that CzcCBA and CusCBA export their substrates *in vivo* mainly from the periplasm through the outer membrane to the outside (30, 51–54). Metal-binding sites of CzcA in the cytoplasm and CzcB in the periplasm might be involved in flux control that prevents export of zinc by CzcCBA under conditions of low zinc availability (55, 56). The periplasmic CzcI protein quenches CzcCBA activity with respect to the essential minor bioelements zinc and cobalt, but not for the toxic-only cadmium (22). CzcI thus prevents an overactive efflux of essential periplasmic zinc and cobalt ions at low concentrations of these ions, allowing the presence of the CzcCBA transmembrane efflux complex under these conditions. CzcCBA has not to be degraded and stands ready in case of a sudden increase in the cobalt, zinc, and cadmium concentrations. However, the zinc importer ZupT is needed to compete with CzcCBA for periplasmic zinc ions. That way, ZupT guarantees zinc import despite the presence of CzcCBA, which explains why cells cannot keep CzcCBA when ZupT is absent.

### The products of the *czcDRSE* region control expression of *czcN* and of *czcP*.

The *czcP* gene encodes a P_IB4_-type zinc-exporting ATPase (30, 57, 58) that exports loosely bound cytoplasmic zinc ions with a high transport rate, while ZntA effluxes firmly bound ions with a lower transport rate. CzcP thus cannot provide zinc resistance without ZntA or one of the other two P_IB2_-type ATPases CadA and PbrA in *C. metallidurans* but is able to enhance resistance mediated by ZntA. The *czcP* gene is expressed only on a low level in nonchallenged cells but upregulated under metal stress (Table 2, Fig. 2, and Fig. S4) (30). A transcriptional start site is located 41 bp upstream of *czcP* and is not RpoD dependent (29).

Expression from *czcNp* and *czcPp* strictly and exclusively depends on the response regulator CzcR and on Zn(II) as inducer (Tables 1 and 2, Fig. 2, and Fig. S4 and S5) (26, 30). Missing upregulation in a Δ*czcR* mutant can be complemented with *czcR* in *trans* on a plasmid. CzcR binds to the respective promoter regions. While *czcNp* contains one binding site, *czcPp* possesses two (27, 30). This agrees with a maximum level of expression from *czcPp* at 150 μM Zn(II) (Fig. 2) compared to a maximum level reached for *czcNp* at 500 μM (Fig. 4). This indicates that a cooperative effect of two CzcR dimers bound to *czcPp* may activate *czcP* expression at lower zinc concentrations than one CzcR dimer bound to *czcNp*. Rapid export of loosely bound cytoplasmic Zn(II) by CzcP is needed at lower zinc concentrations than the function provided by CzcN.

### Cross-talk between two-component regulators embeds the Czc system into metal homeostasis of its host.

A cross talk between the response regulators YedW and CusR in E. coli (59), CopRs and CzcR in Pseudomonas stutzeri (60), and other response regulators (61) has been shown. In *C. metallidurans*, the response regulators closely related to CzcR (Fig. S3) are not able to substitute for CzcR. Instead, CzcS_2_ and AgrS converge on CzcR, which is reminiscent of the histidine kinases RocS_1_ and RocS_2_ that act on the response regulator RocA_1_ in Pseudomonas aeruginosa to control expression of the *cupC* gene involved in copper resistance. Both sensors also act on the response regulator RocA_2_ to repress expression of the *mexAB-oprM* genes, which encode a transenvelope efflux system for organic substances instead of metal ions as the substrate (62).

CzcR_2_S_2_ might control expression of the recessive *czc_2_* determinant, more precisely the genes *czcI_2_C_2_.* These genes and *czcR_2_S_2_* are expressed under RpoD control, *czcI_2_C_2_* from a 134-bp common promoter region but in the opposite direction of transcription from *zntA* (29). Expression of *zntA* is activated by the MerR-type regulator ZntR (22), and the RpoD-dependent transcriptional start site is located 12 bp upstream of *zntA* (29). The transcriptional start site of *czcI_2_*, which is also RpoD dependent, is directly at the 5′ end of *czcI_2_*, indicating lmRNA-specific translation initiation (leaderless mRNA, [63]). This leaves sufficient distance between the promoters to allow simultaneous binding of ZntR and CzcR_2_ so that the RpoD-dependent RNA polymerase holoenzymes attracted to both promoters by both activators may not automatically interfere with each other. On the other hand, this also cannot be excluded.

CzcI_2_ interferes with CzcCBA, similar to CzcI, but in a slightly different manner (22). Expression of *czcC_2_* is strongly upregulated by metals (15). When *czcBA* fragments with Δ*czcC* deletions were expressed in *trans* in the plasmid-free strain AE104, this strain maintained zinc resistance and the ability to efflux zinc compared to a strain expressing *czcCBA* (64). Since an outer membrane factor such as CzcC is an essential part of the transenvelope efflux system (65–68), CzcC_2_ was probably able to substitute for CzcC in these experiments. Because cadmium and cobalt resistance was not maintained, CzcC_2_ may have a higher selectivity for zinc over cobalt and cadmium than CzcC so that an upregulation of *czcI_2_C_2_* may be an advantage under conditions of high zinc availability.

A spontaneous mutation in *agrRS* results in increased silver resistance of *C. metallidurans* (32). The *agrRS* genes are transcribed in the opposite direction from the *agrABC* genes for a transenvelope efflux system from a common promoter region. Two promoters 39 bp and 76 bp upstream of *agrRS* are non-RpoD dependent, but the promoter for *agrABC* depends on the housekeeping sigma factor (29). It is located 45 bp within *agrA* so that the 5′ end of the gene might have been misannotated. Other promoters are far upstream of *agrABC* within *agrRS* and might cause an antisense effect, as has been observed for many transcriptional events in *C. metallidurans* (29). The RND protein AgrA is related neither to the metal-transporting RND proteins of the HME-RND protein family such as CzcA or CusS nor to an AcrB-like transporter for organic substances (Fig. S8). It clearly shows the conserved EN motif at the end of a transmembrane alpha-helix that is important for proton transport. RND proteins involved in transport of divalent transition metal cations possess within this alpha-helix a DFG motif followed by a conserved aspartate 3 positions downstream, which is essential for proton transport (48). Transporters for monovalent cations exhibit an AVG instead of the DFG. In RND proteins for organic substance, an AIG is followed 3 positions downstream by a double aspartate instead of just one (54, 69). AgrA also has the DD signature of organic substrates plus an AVG upstream and a potential metal-binding site, HHRE, downstream of the EN motif in a cytoplasmic part of the RND protein, reminiscent of metal-binding sites in CusAs, CzcA, and SilA from *C. metallidurans* (Fig. S8). AgrA shows hybrid features of an organic and a metal cation transporter. It may transport a metal complex, but an upregulation of *agr* genes under different conditions of metal availability has not yet been observed (15). The most important contribution of *agr* is thus the quenching of CzcR activation under low-zinc conditions.

Signaling by two-component regulatory systems accelerates with HK expression but decelerates with RR expression (25). Expression of *czcS* and of *czcS_2_* is upregulated when metal availability changes but not that of *czcR* (Table S1). Since transcription of *czcDRS* and of *czcE* is under the control of one or two non-RpoD sigma factors and CzcD and CzcE interact with CzcS, a complicated network is in control of the expression of *czcN* and *czcP* (Fig. S7).

### Conclusion.

Together, these data demonstrated a cross talk between AgrRS, CzcR_2_S_2_, and CzcRS involved in control of the *czc* promoters *czcNp* and *czcPp* via CzcR. This cross talk probably used the periplasmic zinc concentration as a signal to regulate expression of *czcP* and *czcN*. In the presence of CzcS, AgrRS and CzcR_2_S_2_ quenched activation of CzcR at low zinc concentrations, with AgrRS being more important than CzcR_2_S_2_. In the absence of CzcS, both cross-talking systems mediated activation of CzcR, albeit after a lag phase. For *czcP-lacZ* in a single-copy environment, the two cross-talking systems contributed equally with an maximum of activation at 100 μM Zn(II) compared to 150 μM Zn(II) in the parent. For *czcNp-lacZ* on a plasmid with a higher copy number, CzcR_2_S_2_ contributed more to activation of CzcR in the absence of CzcS than AgrRS; the maximum was at 200 μM Zn(II) compared to 500 μM in the parent.

## MATERIALS AND METHODS

### Bacterial strains and growth conditions.

Plasmids and *C. metallidurans* strains are provided in Table S3 in the supplemental material. Tris-buffered mineral salts medium (2) containing 2 g sodium gluconate/L (TMM) was used for *C. metallidurans* under aerobic conditions at 30°C.

### Genetic techniques.

Standard molecular genetic techniques were used (70, 71). For conjugative gene transfer, overnight cultures of donor strain E. coli S17/1 (72) and of the *C. metallidurans* recipient strains grown at 30°C in Tris-buffered medium were mixed (1:1) and plated onto nutrient broth agar. After 2 days, the bacteria were suspended in TMM, diluted, and plated onto selective medium as previously described (70).

Plasmid pECD1002, a derivate of plasmid pCM184 (73), was used to construct deletion mutants in *C. metallidurans*. These plasmids harbor a kanamycin resistance cassette flanked by *loxP* recognition sites. Plasmid pECD1002 additionally carries alterations of 5 bp at each *loxP* site. Using these mutant *lox* sequences, multiple gene deletions within the same genome are possible without interference by secondary recombination events (74, 75). Fragments of 300 bp upstream and downstream of the target gene were amplified by PCR, cloned into vector pGEM-T Easy (Promega), sequenced, and further cloned into plasmid pECD1002. The resulting plasmids were used in a double-crossover recombination in *C. metallidurans* strains to replace the respective target gene with the kanamycin resistance cassette, which was subsequently also deleted by transient introduction of *cre* expression plasmid pCM157 (73). Cre recombinase is a site-specific recombinase from the phage P1 that catalyzes the *in vivo* excision of the kanamycin resistance cassette at the *loxP* recognition sites. The correct deletions of the respective transporter genes were verified by Southern DNA-DNA hybridization. For construction of multiple deletion strains, these steps were repeated. The resulting mutants carried a small open reading frame instead of the wild-type gene to prevent polar effects.

### β-Galactosidase assay and *lacZ* reporter constructions in *C. metallidurans*.

To construct reporter operon fusions, a respective promoter region was cloned together with the *lacZ* gene in plasmid pVDZ′2 as described before (26). Alternatively, the *lacZ* reporter gene was inserted downstream of *czcP*. This was done by single crossover recombination in *C. metallidurans* strains. A 300- to 400-bp PCR product of the 3′-end region of the respective target gene was amplified from total DNA of strain CH34, and the resulting fragments were cloned into plasmid pECD794 (pLO2-*lacZ*) (30). The respective operon fusion cassettes were inserted into the open reading frame of the target gene by conjugation and single crossover recombination. *C. metallidurans* cells with a *lacZ* reporter gene fusion were cultivated as a preculture in TMM containing 1.5 g L^−1^ kanamycin at 30°C and 250 rpm for 18 h, diluted to a turbidity of 30 Klett units into fresh medium, and incubated with shaking at 30°C for 3 to 4 h until a cell density of 60 Klett units was reached. This culture was distributed into sterile 96-well plates (Greiner Bio-One, Frickenhausen, Germany). After addition of metal salts, incubation in the 96-well plates was continued for 3 h at 30°C in a neoLab DTS-2 shaker (neoLab Migge Laborbedarf, Heidelberg, Germany). The turbidity at 600 nm was determined in a Tecan Infinite 200 Pro reader (Tecan, Männersdorf, Switzerland), and the cells were sedimented by centrifugation at 4°C for 30 min at 4,500 × *g*. The supernatant was discarded, and the cell pellets were frozen at −20°C. For the enzyme assay, the pellet was suspended in 190 μL Z buffer (60 mM Na_2_HPO_4_, 40 mM NaH_2_PO_4_, 10 mM KCl, 1 mM MgSO_4_, 50 mM beta-mercaptoethanol), and 10 μL permeabilization buffer was added (6.9 mM cetyltrimethylammonium bromide [CTAB], 12 mM sodium deoxycholate). The suspension was incubated with shaking at 30°C, and 20 μL ONPG solution (13.3 mM *ortho*-nitrophenyl-beta-d-galactopyranoside in Z buffer without beta-mercaptoethanol) was added. Incubation was continued with shaking in a neoLab DTS-2 shaker at 30°C until the yellow color of *o*-nitrophenol was clearly visible and stopped by addition of 50 μL 1 M Na_2_CO_3_. The extinction at 420 nm and 550 nm was measured in a Tecan Infinite 200 Pro reader. The activity was determined as published previously (76) with a factor of 315.8 μM calculated from the path length of the 96-well plate and the extinction coefficient of *o*-nitrophenol: activity = 315.8 μM × [*E*_420_ − (1.75 × E_550_)]/reaction time.

Specific activity was activity divided by the cellular dry mass as published previously (76).

For the time-dependent beta-galactosidase assay, at a cell density of 60 Klett units, metal salts were added up to various final concentrations into tubes and the cells were incubated with shaking for a further 3 h. The specific beta-galactosidase activity was acquired in permeabilized cells as published previously with 1 U defined as the activity forming 1 nmol of *o*-nitrophenol per min at 30°C: activity U = 355.6 × *E*_420_/reaction time (76–78).

### RNA isolation and qRT-PCR.

Total RNA was isolated, and the RT reaction was performed as previously described (46). To exclude experimental artifacts resulting from DNA contaminations, only RNA preparations that did not generate products in a PCR with chromosomal primers without a previous RT reaction were used. As an endogenous control, *rpoZ* was used. A no-template control was performed under identical conditions as for the target genes. An average for two different cDNAs as well as an average for two independent biological examples was calculated. For normalization, transcript levels of *rpoZ* were used.

### TSS determination.

RNA was prepared from *C. metallidurans* CH34 cells cultivated in TMM (Tris-buffered mineral salts medium with 2 g/L gluconate as the carbon source) for three independent biological repeats in the presence and absence of a metal ion mix (33), respectively. The composition of this metal cation mix was modified from the published version for a better representation of the individual toxicity of the respective cation. The total metal ion concentration of the metal ion mix used for *C. metallidurans* was 3.347 mM. The 0.1 M stock solution of the metal ion mix contained 13.785 mM Zn(II), 7.215 mM Cu(II), 44.85 mM KH_2_AsO_4_, 11 μM Hg(II), 582 μM K_2_CrO_4_, 439 μM Cd(II), 22.741 mM Ni(II), and 10.376 mM Co(II).

RNA-Seq was performed by Vertis Biotechnology AG (Freising, Germany) using a Cappable-seq protocol for TSS determination (79). The TSSs were trimmed and mapped to the reference genomes CP000352 (chromosome), CP000353 (chromid, also named “megaplasmid”), CP000354 (plasmid pMOL30), and CP000355 (pMOL28), and potential TSSs were annotated as peaks using program tools made available by Laurence Ettwiller (New England Biolabs; https://github.com/Ettwiller/TSS). Since the number of control reads was small compared to the TSS reads, TSSs were calculated without using the control reads. The *n_io_* value was the number of reads at position *i* in orientation *o*, and *N* was the total number of mapped reads. The RRS*_io_* value for each position and orientation was the reads per million and was defined as RRS*_io_* = (*n_io_*/*N*) × 10^6^ for TSS determination and control. For each TSS, the score was RRS*_io_*_TSS/RRS*_io_*_control. For the TSS determination, a cutoff value of RRS*_io_* of 5 and a cluster value of 5 were used, the latter defining the size in base pairs of the upstream and downstream region used for clustering conditions. Only TSSs that appeared in all three biological repeats and had a score of 10 were further considered. Promoter sequences per TSS were extracted as sequence regions 290 to 110 bp around the TSS position on the respective replicon (GenBank accession numbers CP000352.1, CP000353/NC_007974.2, CP000354/NC_007971.2, and CP000355/NC_007972.2) and according to the strand orientation of the TSS. From the resulting database (unpublished data), the mean scores for TSS_74141+4 upstream of *czcN* in RNA from metal-challenged cells, nonchallenged cells, and cells treated with 50 μM EDTA were selected.

### Statistics.

Student’s *t* test was used, but in most cases the distance (*D*) value has been used several times previously for such analyses (80–82). It is a simple, more useful value than Student’s *t* test because nonintersecting deviation bars of two values (*D* > 1) for three repeats always mean a statistically relevant (≥95%) difference provided the deviations are within a similar range. At *n* = 4, significance is ≥97.5%, at *n* = 5, it is ≥99% (significant), and at *n* = 8, it is ≥99.9% (highly significant).
