# Supplementary material for: Interplay between Two-Component Regulatory Systems Is Involved in Control of Cupriavidus metallidurans Metal Resistance Genes
Source: J Bacteriol. 2023 Mar 9;205(4):e00343-22. doi: 10.1128/jb.00343-22 (PMC10127602; doi:10.1128/jb.00343-22)
Supplement: Supplemental file 1 — Tables S1 to S3 and Fig. S1 to S8. Download jb.00343-22-s0001.pdf, PDF file, 2.3 MB [file jb.00343-22-s0001.pdf]

Supplementary material

Supplementary Table S1. Expression and regulation of two-component regulatory systems in *C. metallidurans*[a]

Sensory Histidin Kinase					Response Regulator				
Rmet	Gene	NP.	Res.	Cop.	Rmet	Gene	NP.	Res.	Cop.
**5977**	**CzcS**	**22**	**46**	**CH34**	**5978**	**CzcR**	**39**	**1**	**CH34**
1752	*AgrS*	*7*	*2*	*NF/NF*	*1751*	*AgrR*	*16*	*3*	*NF/NF*
**4466**	**CzcS$_2$**	**8**	**10**	**NF/57**	**4465**	**CzcR$_2$**	**16**	**8**	**33/19**
**5673**	**CopS$_2$**	**6**	**20**	**NF/NF**	**5672**	**CopR$_2$**	**8**	**29**	**NF/160**
**6110**	**CopS$_1$**	**14**	**5**	**CH34**	**6111**	**CopR$_1$**	**18**	**10**	**CH34**
5129		*24*	*3*	*NF/NF*	*5130*		*49*	*4*	*NF/NF*
3015	***HmzS***	***17***	***22***	***NF/NF***	***3016***	***HmzR***	***37***	***56***	***NF/NF***
4830	QseC	45	4	111/NF		QseB	111	3	244/259
**5797**		**44**	**3**	**486/129**	**5798**		**81**	**6**	**105/170**
**4977**	**MmfS**	**1**	**3**	**NQ/NQ**	**4978**	**MmfR**	**0**	**24**	**NQ/NQ**
**5327**	**ZneS**	**7**	**5**	**38/95**	**5326**	**ZneR**	**19**	**7**	**1015/431**
0464		37	3	115/NF	0465		102	2	111/92
3536	*PhhS*	*28*	*2*	*NF/NF*	*3535*	*PhhR*	*143*	*2*	*207/216*
3935		11	2	NF/63	3934		22	2	NF/NF
1626		19	2	40/NF	1627		69	3	59/NF
3584		20	1	779/664	3585		84	2	237/152
5322	***ZniS***	***14***	***6***	***NF/NF***	***5323***	***ZniR***	***45***	***4***	***28/73***
5332	ZneS$_2$	23	3	17/NF	5331	ZneR$_2$	32	4	289/NF

The two-component regulatory systems in the CusS/CzcS/QseC clusters (Suppl. Fig. S2) are listed with the colors corresponding to those in the figure. The light green field indicates association with an active metal resistance determinant, a light red field with an inactivated determinant. The NPKM value (NP.)(1) and Response value (Res.)(2) are indicated. Res. "Response" is the ratio of the largest up-regulation (Q_{max}) of a gene in the data base of gene array results divided by the smallest up- or down-regulation (Q_{min}) and illustrates the range of the regulatory response. Responsive systems (Response of the kinase gene or the regulator gene ≥ 5) is shown in bold-faced letters. Since up- and down-regulations Q were only counted if Qmax ≥ 2 and Qmin ≤ 0.5 corresponding to Qmax/Qmin ≥ 4, a threshold of 5 for the "Response" was selected. Moreover, the copy number of the proteins (Cop.) is indicated in the plasmid-free strain AE104 and its Δ*zupT* mutant which suffers some disturbance in zinc homeostasis: CH34, only in the wild type strain with both plasmids; NF, not found in the proteome and respective proteins in italics; NQ, found but not quantified. Data from (3). Deviations and reproductions are given in the cited references. A former name of *C. metallidurans* was "*Ralstonia metallidurans*" (4-6). Since the names of the *C. metallidurans* genes have been changed many times, the "Rmet" locus tags are provided as easy reference.

Supplementary Table S2. Polynomial fitting of the results of the expression of *czcNp-lacZ* in plasmid pVDZ'2 in ∆*czcS* single, double and triple mutant strains in comparison with a fitting of the *czcPp-lacZ* data to a binomial function[a].

Strain	Coeff.	a, (U/mg)	b, (U/mg/min)	c, (U/mg/min^2) x 1000
czcNp-lacZ				
DN179∆(*czcS)*	99.5%	1380±114	-4.83±2.40	53.9±11.3
DN575∆(*czcS* ∆*agrRS*)	98.9%	1312±90	-5.35±2.62	42.8±13.4
DN576∆(*czcS* ∆*czcR$_2$S$_2$*)	98.5%	1435±98	-5.39±2.52	32.7±11.8
DN577, triple mutant	98.2%	1286±75	-3.70±1.86	18.0±8.4
czcPp-lacZ				
DN179∆(*czcS)*	99.2%	20.3±4.1	0.267±0.081	0.470±0.322
DN575∆(*czcS* ∆*agrRS*)	98.6%	22.6±3.7	0.0495±0.0737	0.809±0.294
DN576∆(*czcS* ∆*czcR$_2$S$_2$*)	99.1%	23.6±3.6	0.0966±0.0719	0.840±0.287
DN577, triple mutant	99.9%	26.7±0.7	-0.00483±0.0146	0.878±0.058

[a]The result are shown in Fig. 4B. Fitting was done with profit 7.0.19 (www.quansoft.com)

Supplementary Table S3. Strains and primers

Strain	Relevant markers	Reference
	Cupriavidus metallidurans	
AE128	pMOL30	(7)
DN178	pMOL30, Δ*czcR*	(8)
DN179	pMOL30, Δ*czcS*	(8)
DN493	pMOL30, Δ*czcP*	(9)
DN572	pMOL30, Δ*agrRS*	this study
DN573	pMOL30, Δ$czcR_2S_2$	this study
DN574	pMOL30, Δ*agrRS* Δ$czcR_2S_2$	this study
DN575	pMOL30, Δ*czcs* Δ*agrRS*	this study
DN576	pMOL30, Δ*czcS* Δ$czcR_2S_2$	this study
DN577	pMOL30, Δ*czcS* Δ*agrRS* Δ$czcR_2S_2$	this study
plasmids		
pDNA364	*czcI-lacZ* in pVDZ´2	(10)
pDNA358	*czcN-lacZ* in pVDZ´2	(10)
pECD1028	*czcP-lacZ* in pLO2	(9)

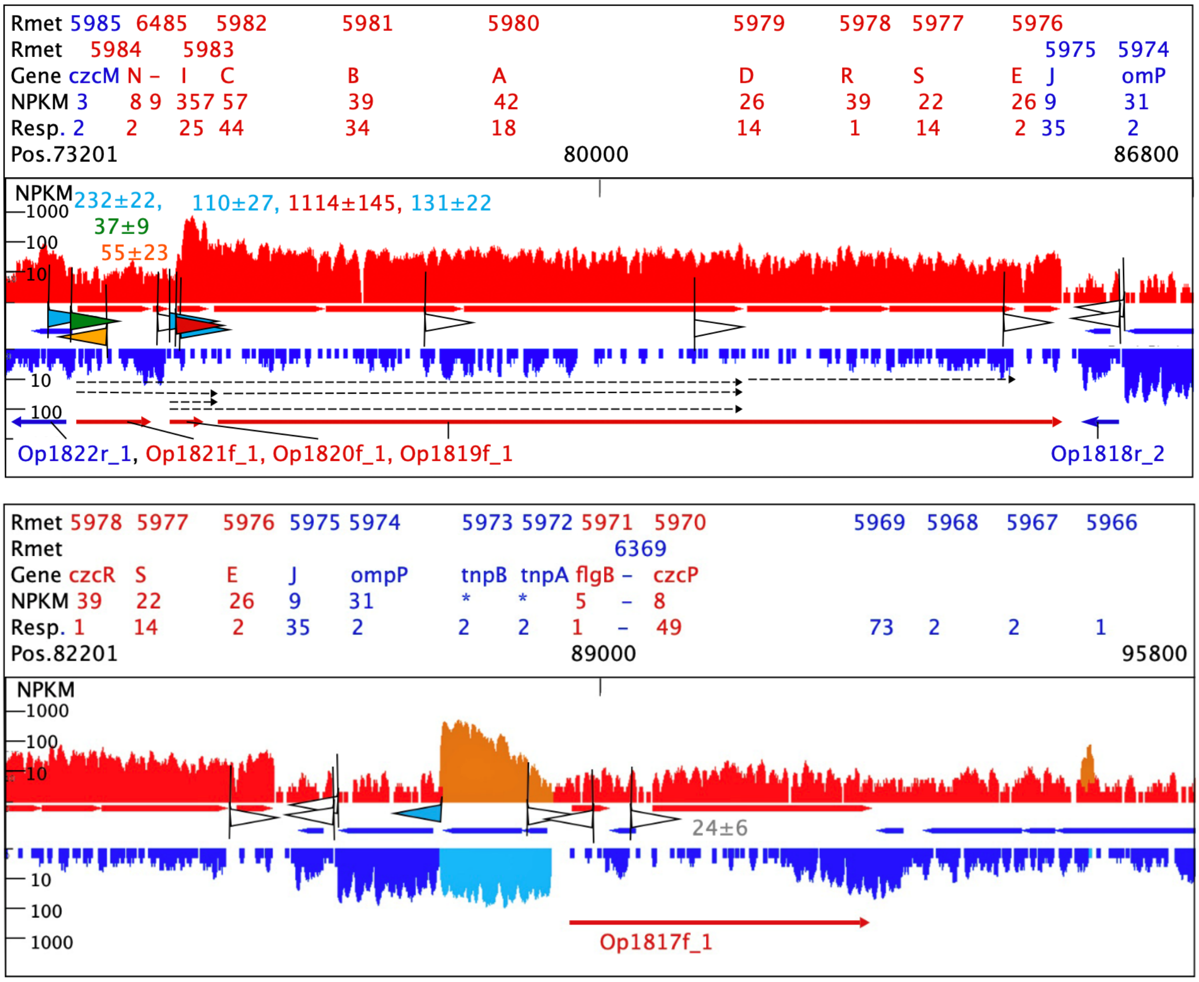

Supplementary Fig. S1. Map of the cobalt-zinc-cadmium resistance determinant *czc* on plasmid pMOL30. The map shows the first (top) and second (bottom) part of the *czc* determinants in the indicated regions with NPKM values (nucleotide activities per kilobase of exon model per million mapped reads, a measure of RNA abundance) on one DNA strand (red) or the other direction of transcription (blue). Regions in light blue and orange indicate transcripts from multiple identical DNA regions in *C. metallidurans*. Above are the Rmet locus and gene names, the mean NPKM and response values (2). Transcriptional start sites (TSSs, flags) are indicated with the corresponding TSS score, white if score < 50, red shades for strong (>1000, red) or medium (50-1000, orange) transcription initiation from RpoD promoters (medium or strong RpoD score), blue shades accordingly if not or only weakly associated to the RpoD model (11). Transcripts (8) and operons (1) are also indicated in the transcript map. With one exception, TSSs with scores < 50 (white arrows) were shown without TSS scores. Both maps have been published (11). The real *czcNp* promoter has only be recently identified 70 bp upstream of *czcN* (green flag) because it had been previously obscured by the repeated changes in the annotation of *czcN* and the *C. metallidurans* genome. A former name of *C. metallidurans* was "*Ralstonia metallidurans*" (4-6). Since the names of the *C. metallidurans* genes have been changed many times, the "Rmet" locus tags are provided as easy reference.

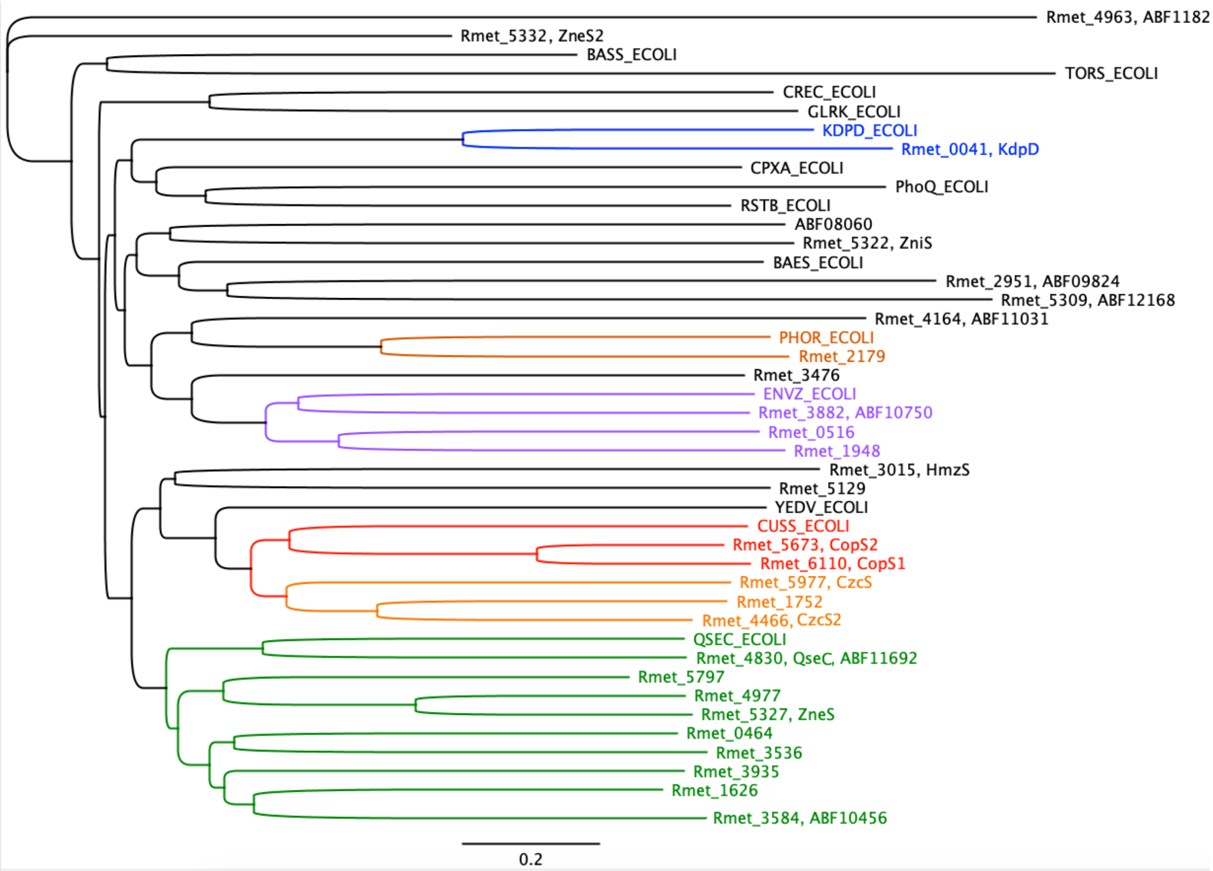

Supplementary Figure S2. Predicted histidine kinases in the genome of *C. metallidurans* compared to some in *E. coli*. Multiple alignment performed with Geneious (https://support.geneious.com), cost matrix Blosum62, open gap penalty 12, gap extension penalty 3, 2 refinement iterations. Different colors were used to label the KpdD-, PhoR-, EnvZ-, CusS-, CzcS-, and QseC-clusters, respectively. BarA and UhpB from *E. coli* are not shown in this alignment and cluster with TorS and the KdpD-like proteins outside of the cluster of metal-sensing histidine kinases.

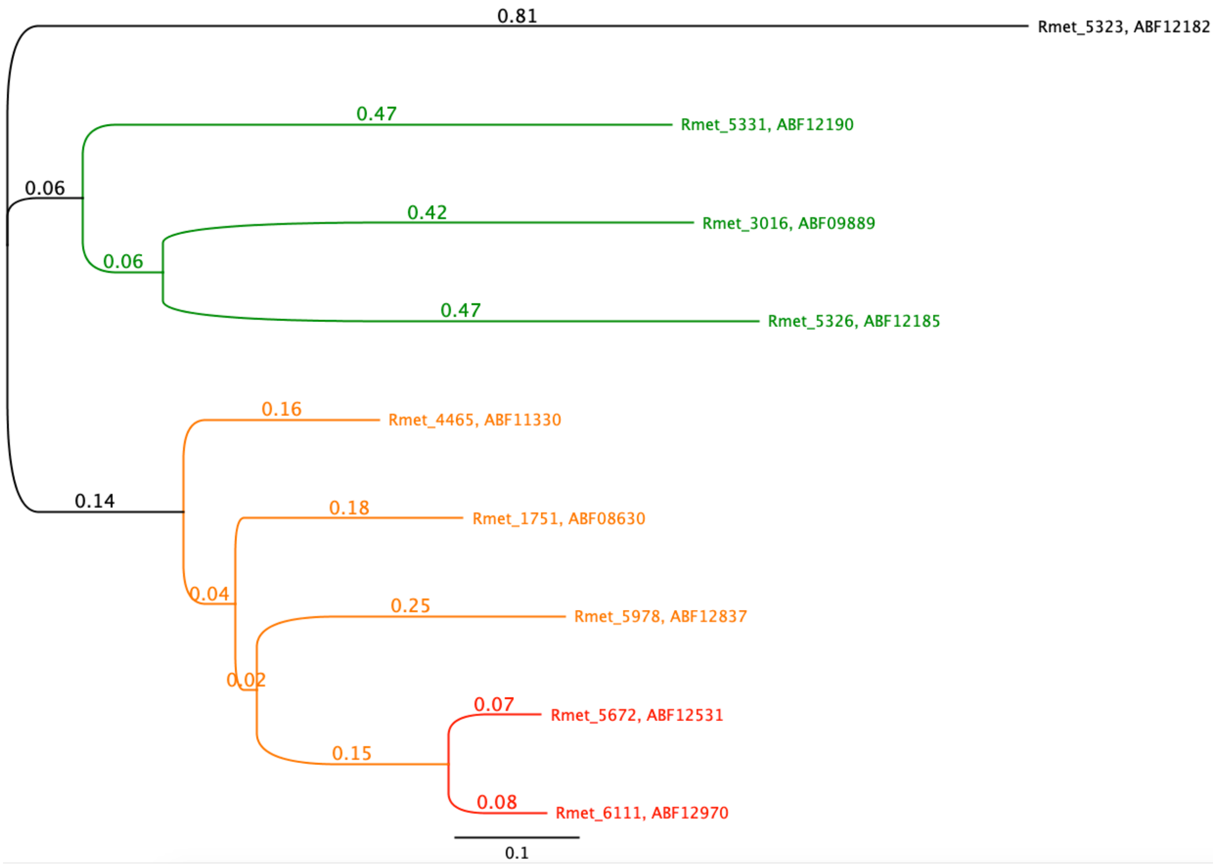

Supplementary Figure S3. Comparison of predicted response regulators. Multiple alignment performed with Geneious (https://support.geneious.com), cost matrix Blosum62, open gap penalty 12, gap extension penalty 3, 2 refinement iterations. Different colors were used to label the CopR-cluster (red, Rmet_5672, Rmet_6111), the CzcR-cluster (orange, CzcR = Rmet_5978, AgrR = Rmet_1751, HmuR = Rmet_4465 = $CzcR_2$), ZniR (black, Rmet_5323), and the ZneR-Cluster (green; ZneR = Rmet_5326, $ZneR_2$ = Rmet_5331, HmzR = Rmet_3016).

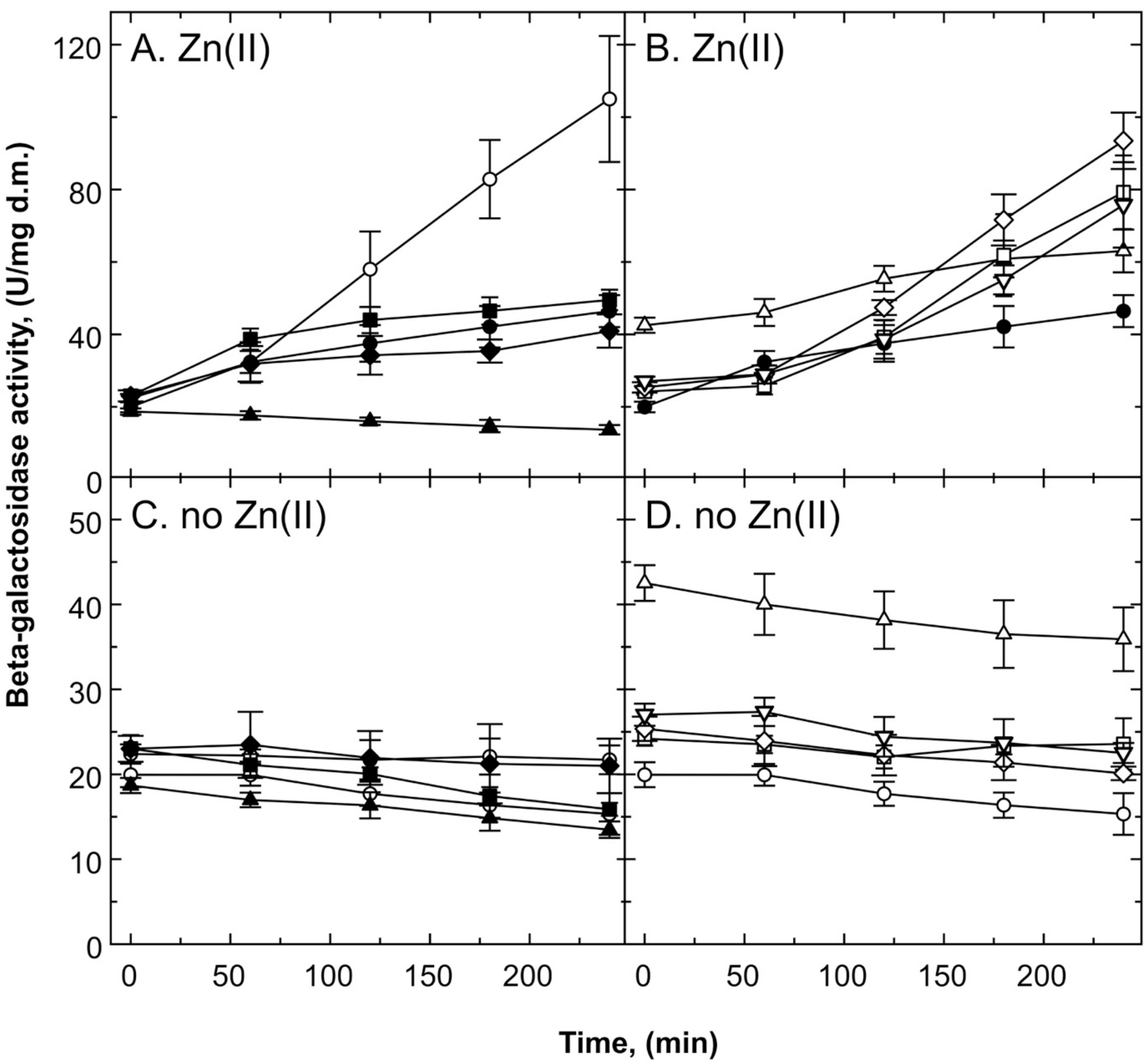

Supplementary Figure S4. Time-dependent increase in expression of *czcP-lacZ* on plasmid pMOL30. The cells were incubated with 200 µM Zn(II) (Panels A, B) or without Zn(II) (Panels C, D) and the specific beta-galactosidase activity was measured after the indicated time. The strains carrying the *czcP-lacZ* fusion derived from AE128 parent (closed circles,●), DN178 (Δ*czcR,* closed triangles,▲), DN179 (Δ*czcS*, open circles,❍), DN572 (Δ*agrRS*, closed squares,■), DN573 (Δ$czcR_2S_2$, closed diamonds,◆), DN574 (Δ*agrRS* Δ$czcR_2S_2$, open triangles,△), DN575 (Δ*czcS* Δ*agrRS,* open squares,☐), DN576 (Δ*czcS,* Δ$czcR_2S_2$, open diamonds, ◇) or DN577 (Δ*czcS* Δ$czcR_2S_2$ Δ*agrRS*, open inverted triangles,▽). N = 6, deviations shown.

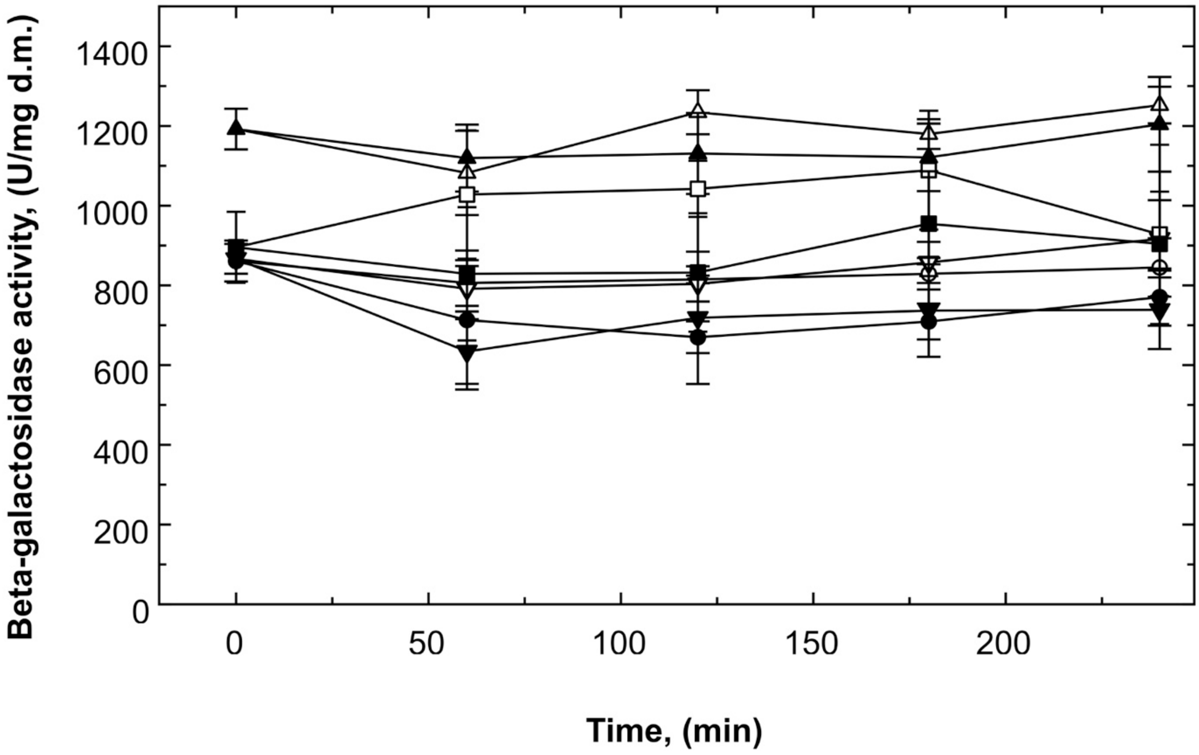

Supplementary Figure S5. Time-dependent increase in expression of *czcNp-lacZ* on vector plasmid pVDZ'2, negative control. The cells were incubated with 750 µM Zn(II) (closed symbols, ▲,●,■,▼) or without Zn(II) (open symbols, △,❍,☐,▽) and the specific beta-galactosidase activity was measured after the indicated time. One strain carried the *czcNp-lacZ* fusion on vector plasmid pVDZ'2 in strain DN178 (∆*czcR,* triangles,▲, △). The others are negative controls: (i) vector plasmid pVDZ'2 with the *lacZ* gene but without a cloned promoter upstream in strains AE128 parent (circles,●,❍); (ii) in strain DN178 (∆*czcR,* inverted triangles, ▼,▽); and (iii) in strain DN179 (∆*czcS*, squares,■,☐). n = 3, deviations shown.

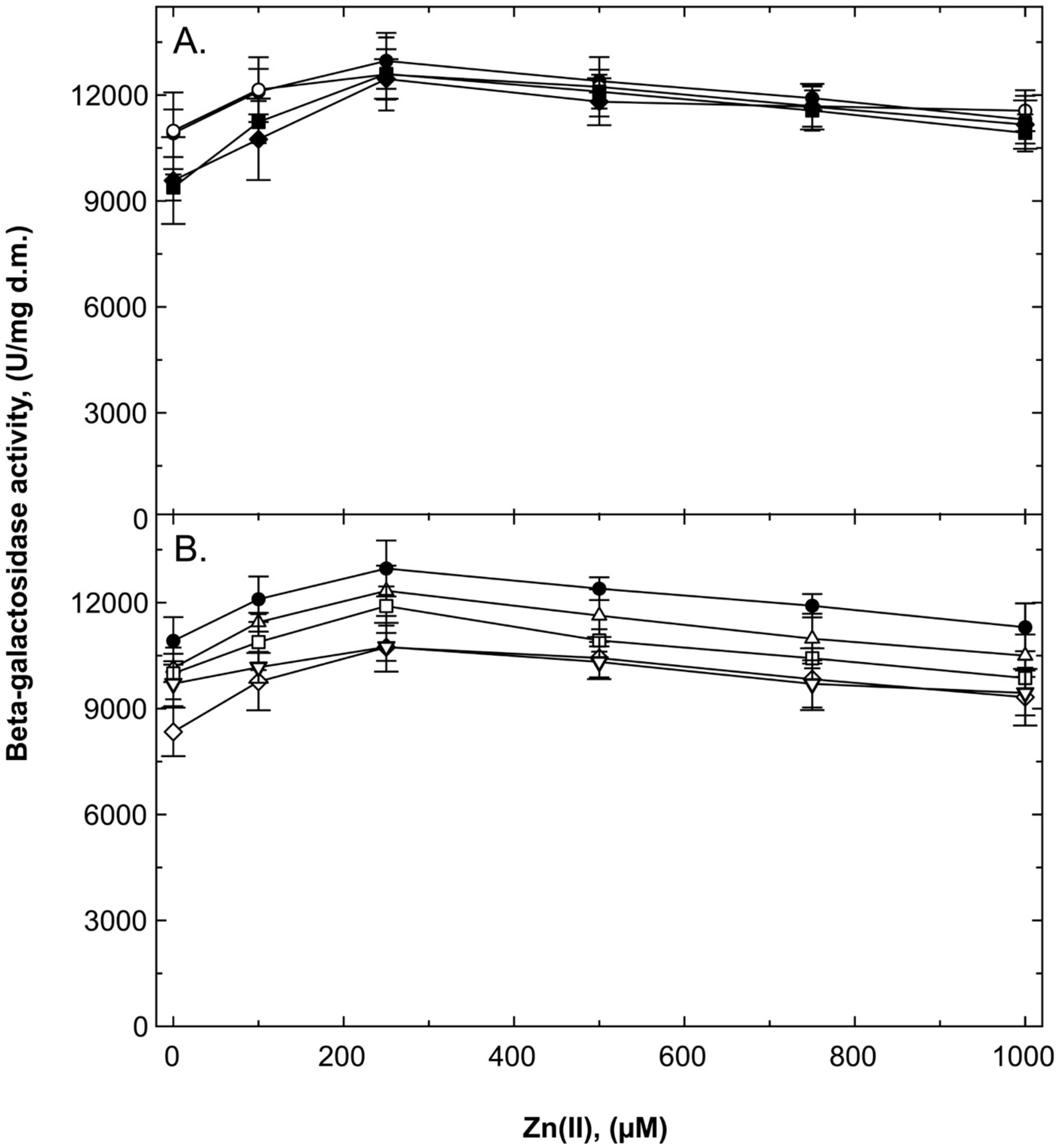

Supplementary Figure S6. Concentration-dependent increase in expression of *czcIp-lacZ* on plasmid pVDZ'2. The cells were incubated with various zinc concentrations for 3 h and the specific beta-galactosidase activity was determined. All strains carrying the *czcIp-lacZ* fusion on plasmid pVDZ'2 in AE128 parent (closed circles,●) in all panels. Panel A, single mutants DN179 (Δ*czcS*, open circles,❍), DN572 (Δ*agrRS*, closed squares,■), DN573 (ΔczcR$_2$S$_2$, closed diamonds,◆). Panel B, DN575 (Δ*czcS* Δ*agrRS,* open squares,☐), DN576 (Δ*czcS,* Δ*czcR$_2$S$_2$,* open diamonds, ◇). Panel C, double mutant DN574 (Δ*agrRS* Δ*czcR$_2$S$_2$*, open triangles,△) and triple mutant DN577 (Δ*czcS* Δ*czcR$_2$S$_2$* Δ*agrRS*, open inverted triangles,▽). N = 3, deviations shown.

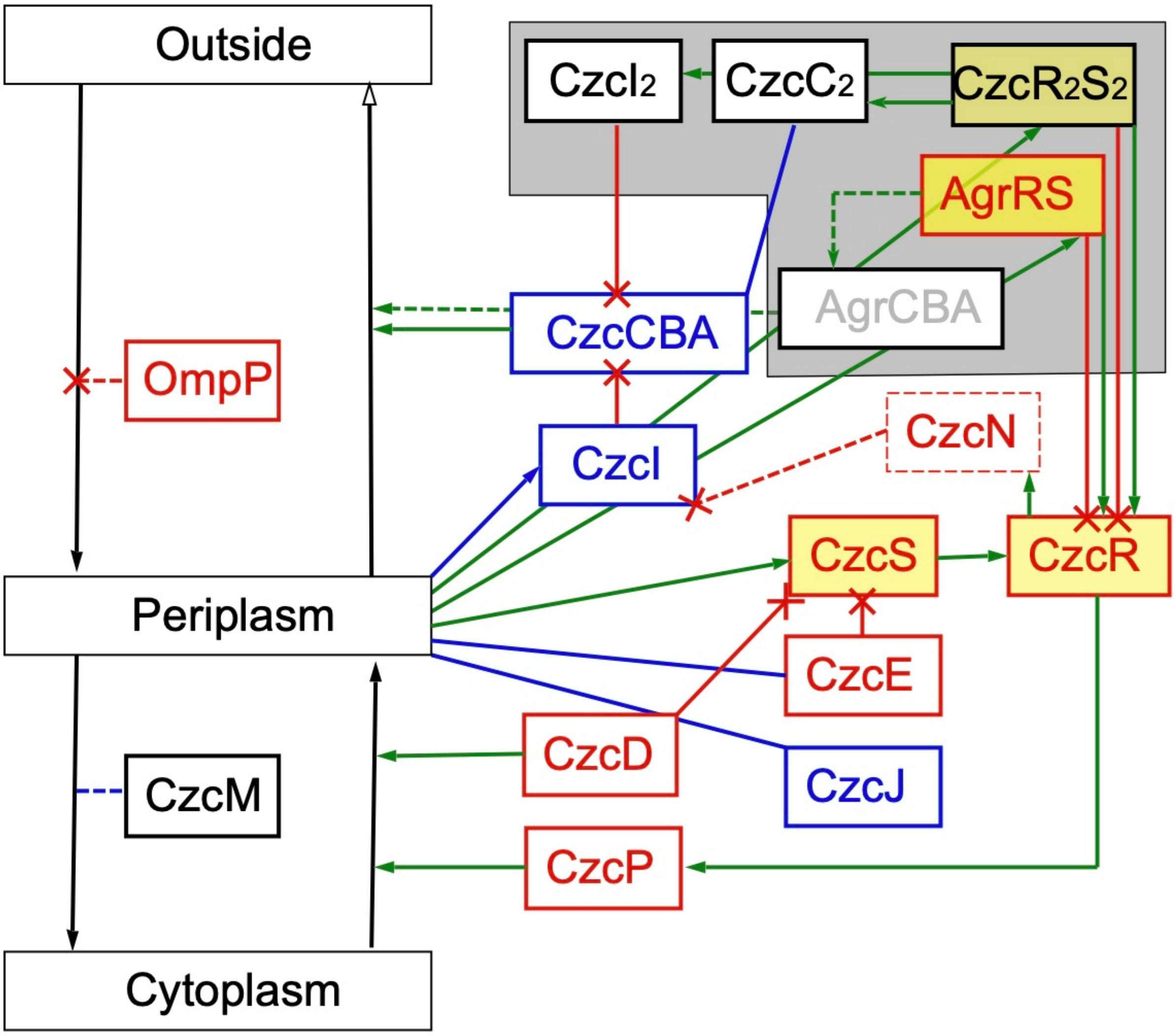

Supplementary Figure S7. Regulatory network surrounding CzcCBA. The periplasmic concentration of the three cations Zn(II), Co(II) and Cd(II) results from an kinetic flow equilibrium formed by (i) import reactions from the outside; (ii) from the cytoplasm (which are inner membrane efflux reactions from the point of view of the cytoplasm); (iii) export reactions from the periplasm to the outside; and (iv) to the cytoplasm (which are uptake reactions as far as the cytoplasm is concerned). Products of the *czc* determinant may decrease uptake from the outside and into the cytoplasm, CzcD and CzcP import into the periplasm from the cytoplasm and export by CzcCBA to the outside. The periplasmic metals interfere with periplasmic metal-binding proteins and the histidine kinase CzcS, which activates the response regulator CzcR. Red lines indicate inhibition, green lines activation, blue lines not known mechanisms, dashed lines speculations. The grey field gives the function of the two-component regulatory systems that interfere with CzcR. Black field surroundings of the components indicate their expression by the housekeeping sigma factor RpoD, red surrounding of non-RpoD sigma factors, blue both. The systems at the focus of this publication are on a yellow field. OmpP may be an outer membrane protein changing import of metal cations across this membrane. CzcM is related to MgtC, which inhibits uptake of phosphate under conditions of magnesium starvation (12), which subsequently may influence metal uptake. The function of the periplasmic protein CzcJ is not yet known.

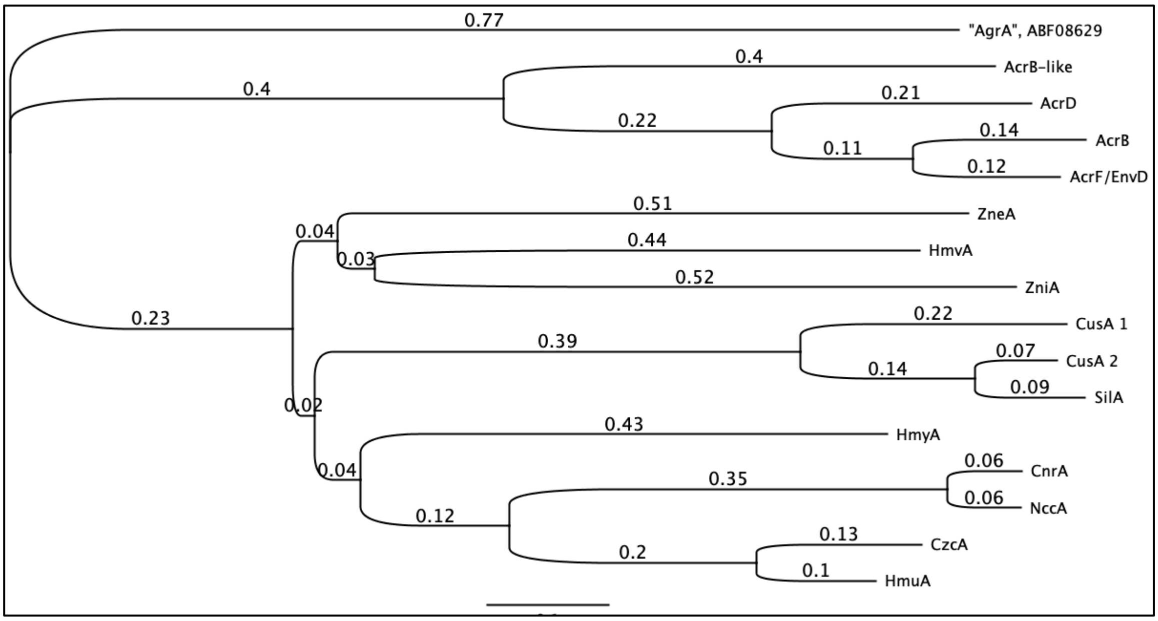


```
AgrA        IALSALGQTLNIMTLGGLALAVGVLVDDATVAIENITHHRE------MSKPLEDA-----
AcrB        AVLAAFGFSINTLTMFGMVLAIGLLVDDAIVVVENVERVMA-------EEGLPPKE-A--
AcrB-like   ACLYAFGLSLNVITLFGVVLAIGILVDDAIVVVENVERIMR-------EEGVDAFT-A--
AcrD        SVLYAFGYSVNTLTMFAMVLAIGLLVDDAIVVVENVERIMS-------EEGLTPRE-A--
AcrF/EnvD   AILAAFGYSINTLTMFGMVLAIGLLVDDAIVVVENVERVMM-------EDKLPPKE-A--
CnrA        IGMNQFHISGNLMSLG--ALDFGLIIDGAVIIVENSLRRLAE-RQHREGRLLTLDERL--
CusA 1      IVMHFQGLNANIMSLGGIAIAVGAMVDAAIVMIENAHKRLEEWQHQHPDATLDNKTRW--
CusA 2      LVMRYQGVNANIMSLGGIAIAVGAMVDAAVVMIENAHKHLERWHADHPGRPLSGDDRW--
CzcA        TGMVNYKISANLMSLG--ALDFGIIIDGAVVIVENCVRRLAHA-QEHHGRPLTRSERF--
HmuA        TGMVSYKISANLMSLG--ALDFGIIVDGAVVIVENCVRRLAHAK-ERLGRPLTRAERF--
HmvA        IGLTWVGIPANLLSLG--AMDFGIIVDGAVIVVENIFKRLGELK---EQQIKDNKARM--
HmyA        IVMNQVGLSANLMSLGGLAIAIGLMVDGSVVVVENAFERLGHA----EKTGLTRTQ-V--
NccA        IGMNQLGISGNLMSLG--ALDFGLIIDGAVIIVENTLRRLAQRQ-HQEGRLLTLRERL--
SilA        LVMRYQGVNANIMSLGGIAIAVGAMVDAAVVMIENAHKHLEHWHVKHPQQELEAQERW--
ZneA        ILMHHFKIPANLLSLG--AIDFGIIVDGAIVVMENILRRREED----AEKELHGRD-I--
ZniA        MILVMLGESANLLSVG--AVDFGIIVDSSVILVENIFRNFQMPLADQQRLLMSRDARAIG
```

Supplementary Figure S8. Relationship of AgrA to other RND proteins. RND-proteins from *C. metallidurans* and *E. coli* were aligned with Geneious (https://support.geneious.com), cost matrix Blosum62, open gap penalty 12, gap extension penalty 3, 2 refinement iterations, distances shown (top). HmuA is synonymous with CzcA2. The multiple alignment below shows the amino acid sequences related to the region from Thr384 to Phe438 of CzcA with the conserved motifs 402-DFG-404, D408 and 415-EN-416 (bold red) characteristically for HME-RND proteins exporting divalent transition metal cations. Those exporting monovalent cations Cu(I) and Ag(I) exhibit instead of "DFG" an "AVG" (blue) while HAE1(13, 14)-RND exporters of organic molecules have an "AIG" (green) followed by a double-D motif (purple) (15, 16). AgrA is special among these proteins. It is not related to HME- or HAE-type RND proteins and combines an "AVG" of a monovalent ion with the double-D of an exporter for organic substances.

References of the Supplement

1. Große C, Poehlein A, Blank K, Schwarzenberger C, Schleuder G, Herzberg M, Nies DH. 2019. The third pillar of metal homeostasis in *Cupriavidus metallidurans* CH34: Preferences are controlled by extracytoplasmic functions sigma factors. Metallomics 11:291-316.
2. Große C, Kohl T, Herzberg M, Nies DH. 2022. Loss of mobile genomic islands in metal resistant, hydrogen-oxidizing *Cupriavidus metallidurans*. Appl Environ Microbiol 88:e02048-21.
3. Herzberg M, Dobritzsch D, Helm S, Baginski S, Nies DH. 2014. The zinc repository of *Cupriavidus metallidurans*. Metallomics 6:2157-2165
4. Vandamme P, Coenye T. 2004. Taxonomy of the genus *Cupriavidus*: a tale of lost and found. Int J Syst Evol Microbiol 54:2285-2289.
5. Vaneechoutte M, Kämpfer P, De Baere T, Falsen E, Verschraegen G. 2004. *Wautersia* gen. nov., a novel genus accommodating the phylogenetic lineage including *Ralstonia eutropha* and related species, and proposal of *Ralstonia* [*Pseudomonas*] *syzygii* (Roberts et al. 1990) comb. nov. Int J Syst Evol Microbiol 54:317-327.
6. Yabuuchi E, Kosako Y, Yano I, Hotta H, Nishiuchi Y. 1995. Transfer of two *Burkholderia* and an *Alcaligenes* species to *Ralstonia* gen. nov.: proposal of *Ralstonia pickettii* (Ralston, Palleroni and Doudoroff 1973) comb. nov., *Ralstonia solanaceaum* (Smith, 1896) comb. nov. and *Ralstonia eutropha* (Davis 1969) comb. nov. Microbiol Immunol 39:897-904.
7. Mergeay M, Nies D, Schlegel HG, Gerits J, Charles P, van Gijsegem F. 1985. *Alcaligenes eutrophus* CH34 is a facultative chemolithotroph with plasmid-bound resistance to heavy metals. J Bacteriol 162:328-334.
8. Große C, Grass G, Anton A, Franke S, Navarrete Santos A, Lawley B, Brown NL, Nies DH. 1999. Transcriptional organization of the *czc* heavy metal homoeostasis determinant from *Alcaligenes eutrophus*. J Bacteriol 181:2385-2393.
9. Scherer J, Nies DH. 2009. CzcP is a novel efflux system contributing to transition metal resistance in *Cupriavidus metallidurans* CH34. Mol Microbiol 73:601-621.
10. Große C, Anton A, Hoffmann T, Franke S, Schleuder G, Nies DH. 2004. Identification of a regulatory pathway that controls the heavy metal resistance system Czc via promoter *czcNp* in *Ralstonia metallidurans*. Arch Microbiol 182:109-118.
11. Große C, Grau J, Große I, Nies DH. 2022. Importance of RpoD- and non-RpoD-dependent expression of horizontally acquired genes in *Cupriavidus metallidurans*. Microbiol Spectr 10: 10.1128/spectrum.00121-22.
12. Bruna RE, Kendra CG, Groisman EA, Pontes MH. 2021. Limitation of phosphate assimilation maintains cytoplasmic magnesium homeostasis. Proc Natl Acad Sci U S A 118.
13. Nies DH. 2003. Efflux-mediated heavy metal resistance in prokaryotes. FEMS Microbiol Rev 27:313-339.
14. Nies DH. 2013. RND-efflux pumps for metal cations, p 79-122. *In* Yu EW, Zhang Q, Brown MH (ed), Microbial efflux pumps: current research doi:ISBN: 978-1-908230-21-8. Caister Academic Press, Norfolk, UK.
15. Goldberg M, Pribyl T, Juhnke S, Nies DH. 1999. Energetics and topology of CzcA, a cation/proton antiporter of the RND protein family. J Biol Chem 274:26065-26070.
16. Tseng T-T, Gratwick KS, Kollman J, Park D, Nies DH, Goffeau A, Saier MHJ. 1999. The RND superfamily: an ancient, ubiquitous and diverse family that includes human disease and development proteins. J Mol Microbiol Biotechnol 1:107-125.
